# TadA reprogramming to generate potent miniature base editors with high precision

**DOI:** 10.1038/s41467-023-36004-2

**Published:** 2023-01-26

**Authors:** Shuqian Zhang, Liting Song, Bo Yuan, Cheng Zhang, Jixin Cao, Jinlong Chen, Jiayi Qiu, Yilin Tai, Jingqi Chen, Zilong Qiu, Xing-Ming Zhao, Tian-Lin Cheng

**Affiliations:** 1 Institute for Translational Brain Research, State Key Laboratory of Medical Neurobiology, MOE Frontiers Center for Brain Science, Institute of Pediatrics, National Children’s Medical Center, Children’s Hospital, Fudan University, Shanghai, China; 2 Department of Pediatrics, Qilu Hospital of Shandong University, Ji’nan, 250012 China; 3 Institute of Science and Technology for Brain-Inspired Intelligence, Fudan University, Shanghai, China; 4 Institute of Neuroscience, State Key Laboratory of Neuroscience, CAS Center for Excellence in Brain Science and Intelligence Technology, Chinese Academy of Sciences, Shanghai, 200031 China; 5 Institutes of Brain Science, State Key Laboratory of Medical Neurobiology, MOE Frontiers Center for Brain Science, Fudan University, Shanghai, 200032 China; 6 State Key Laboratory of Medical Neurobiology, Institutes of Brain Science, Fudan University, Shanghai, China; 7 MOE Key Laboratory of Computational Neuroscience and Brain-Inspired Intelligence, and MOE Frontiers Center for Brain Science, Fudan University, Shanghai, China; 8 National Clinical Research Center for Aging and Medicine, Huashan Hopsital, Fudan University, Shanghai, 200032 China; 9 Songjiang Hospital, Songjiang Institute, Shanghai Jiao Tong University School of Medicine, Shanghai, China

**Keywords:** CRISPR-Cas9 genome editing, Biotechnology, Molecular engineering

## Abstract

Although miniature CRISPR-Cas12f systems were recently developed, the editing efficacy and targeting range of derived miniature cytosine and adenine base editors (miniCBEs and miniABEs) have not been comprehensively addressed. Moreover, functional miniCBEs have not yet be established. Here we generate various Cas12f-derived miniCBEs and miniABEs with improved editing activities and diversified targeting scopes. We reveal that miniCBEs generated with traditional cytidine deaminases exhibit wide editing windows and high off-targeting effects. To improve the editing signatures of classical CBEs and derived miniCBEs, we engineer TadA deaminase with mutagenesis screening to generate potent miniCBEs with high precision and minimized off-target effects. We show that newly designed miniCBEs and miniABEs are able to correct pathogenic mutations in cell lines and introduce genetic mutations efficiently via adeno-associated virus delivery in the brain in vivo. Together, this study provides alternative strategies for CBE development, expands the toolkits of miniCBEs and miniABEs and offers promising therapeutic tools for clinical applications.

## Abstract (web summary)

Hypercompact CRISPR-Cas12f systems have been engineered to generate miniABEs but these have limitations. Here the authors generate Cas12f-derived miniCBEs and develop miniABEs with improved editing and targeting scopes; they use these to correct pathogenic mutations in cell lines and introduce mutations in vivo.

## Introduction

Base editors, established to achieve efficient C-to-T (CBEs) and A-to-G editing (ABEs) by fusing deaminases to nicking/deactivated Cas nucleases (nCas9 or dCas9), are promising therapeutic tools^1–3^. However, Cas9-derived base editors usually are too large to be carried by adeno-associated virus (AAV), the commonly used gene delivery vehicle, thereby limiting potential therapeutic applications.

Recently multiple hypercompact CRISPR or ancestor systems have been identified^4–9^, providing more choices for genome editing via all-in-one AAV delivery system. Hypercompact CRISPR-Cas12f systems have been engineered for functional miniABEs^6–10^. Nevertheless, Cas12f-derived miniABEs are still hindered by modest A-to-G editing activity and restricted editing scopes^7,11^. Moreover, no functional miniCBEs have been reported so far.

Classical ABEs contained evolved tRNA-specific adenosine deaminases from *Escherichia coli* (ecTadA) (evolved ecTadA variant, simplified as TadA*) while CBEs contained cytidine deaminases^2,3,12^. RNA off-targeting of ABEs and Cas9-indenpdent DNA off-targeting of CBEs are attributed to intrinsic properties of adenosine and cytidine deaminases^13–19^, which severely restricted their applications. Deaminase engineering and screening have been extensively exploited^20–27^. However, deaminases with optimized properties remain limited, and further engineering is required. It was noticed that ABEs also displayed C-to-T editing activity within specific sequence contexts, and specific mutations could modulate adenine and cytosine editing activities separately^20,28,29^. For example, P48R mutation in TadA* of ABE7.10 enhanced TC-specific cytosine editing and inhibited adenine editing concurrently. Additionally, it has been reported that domain insertion inside nCas9 also modulates cytosine editing capacity of ABEs^20,28,29^. Therefore, it is possible to reprogram the substrate specificity of ABEs to generate functional CBEs and ACBEs via TadA reprogramming. As conventional CBEs, composed of classical cytidine deaminases and nCas9/dCas9, displayed obvious characteristic Cas9-indenpdent DNA off-targeting^13–19^ while available ACBEs required two deaminase domains leading to larger size, TadA reprogramming represents one promising strategy for the optimization of base editors.

Here we generated a series of Cas12f-derived functional miniABEs and miniCBEs with diversified editing signatures. We further executed mutagenesis screening to reprogram TadA for efficient C-to-T editing, and TadA-reprogrammed miniCBEs displayed potent C-to-T editing, high precision, and minimized off-target effects simultaneously. In proof-of-concept applications, miniABEs, and miniCBEs corrected or installed specific pathogenic mutations in vitro and in vivo efficiently.

## Results

### Development of miniABEs with high editing activities and diversified scopes

**Fig. 1:**
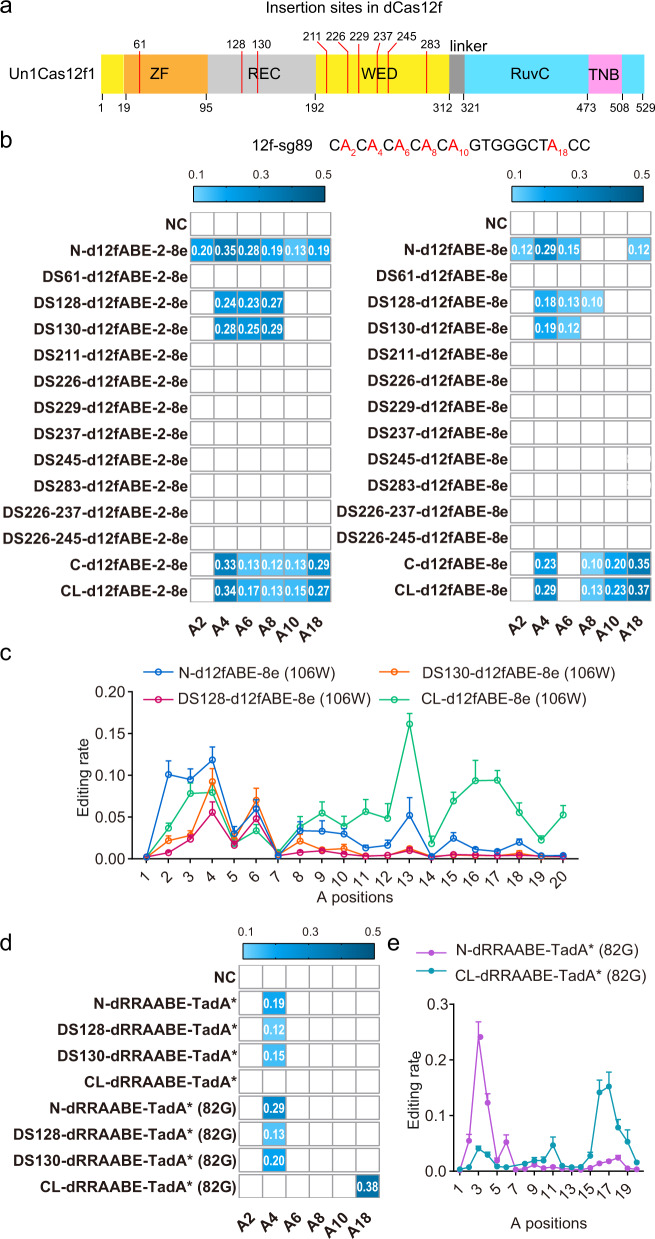
Development of functional d12fABEs with high activities and diversified scopes. **a** Diagram showing Cas12f domain organization and internal fusing sites (red lines) for the generation of potential miniABEs. **b** Heatmaps from left to right showing A-to-G editing frequencies of d12fABEs-2-8e and d12fABEs-8e in blue gradient color. Various potential d12fABEs are generated by fusing TadA-8e monomer (hereafter 8e), dimer (hereafter 2-8e) to N-, C-terminus or internal sites of d12f. Editing activities were evaluated against sgRNA 12f-sg89. **c** A-to-G editing frequencies at each adenine position across 12 endogenous sites were quantified and summarized for N-/CL-/DS128-/DS130-d12fABE-8e (106 W) in blue, orange, violet, and green lines, respectively. **d** Heatmap showing editing activities of d12fABEs-TadA* and d12fABEs-TadA* (V82G) against sgRNA 12f-sg89 in blue gradient color. **e** A-to-G editing frequencies at each adenine position across 12 endogenous sites were quantified and summarized for N-dRRAABE-TadA* (82 G) and CL-dRRAABE-TadA* (82G) in purple and cyan lines, respectively. NC negative control. All data were collected from three independent experiments and presented as mean ± SEM. in histograms. Source data are provided as a Source Data file.

Deaminase insertion inside nCas9 led to altered editing scopes and reduced DNA/RNA off-target effects^20,22,24^. Then flexible regions inside Cas12f and close to non-target single-stranded DNA (ssDNA) were selected as internal docking sites for deaminase fusing^30,31^ (Fig. 1a, Supplementary Table 1, Supplementary Movie). As TadA-8e was one of the most potent adenosine deaminase variant^32^, we generated various potential miniABEs by fusing TadA-8e monomer (hereafter 8e) or dimer (hereafter 2-8e) to N-terminus, C-terminus or selected internal docking sites of nuclease-deactivated Un1Cas12f1 (hereafter d12f) (hereafter d12fABEs-8e and d12fABEs-2-8e, respectively; d12fABEs generated by different fusing strategies were labeled as N-/CL-/DSX-d12fABE, with DSX representing internal docking site X) (Fig. 1a). We chose an engineered single guide RNA (sgRNA) backbone ge4.1 for subsequent experiments^6^. Functional analysis with one sgRNA (12f-sg89) revealed that N-terminus, C-terminus, and internal DS128/130-fusing strategies generated functional d12fABEs with up to 37% editing frequencies (Fig. 1b). We also showed that TadA-8e mutant V106W (hereafter 8e (106 W), 2-8e (106 W)) with reduced RNA off-targeting effects^32^, could generate functional d12fABEs-8e (106 W) and d12fABEs-2-8e (106 W), with comparable editing activities to TadA-8e (Supplementary Fig. 1a-b). Four representative d12fABEs-8e (106 W) were further evaluated across 12 endogenous sites by targeted amplicon sequencing. It was shown that N-d12fABE-8e (106 W) displayed an editing window at A2-A4 (an average of 9.5%~11.9% editing frequency, R of PAM TTTR counted as 0) distal to PAM, similar to reported miniABEs^7,11^ (Fig. 1c). DS128-d12fABE-8e (106 W) and DS130-d12fABE-8e (106 W) displayed editing windows at A4 and A6 with lower editing activities (4.8%~5.6% and 7.0%~9.3%, respectively) (Fig. 1c). CL-d12fABE-8e (106 W) displayed complex editing window, with the highest editing activity at A13 (16.2%) and modest activity at A2-A4, A6, A8-A12 and A15-A18 (3.4–9.4%) (Fig. 1c).

D143R/T147R/E151A mutations (hereafter, RRA) could enhance Cas12f nuclease activity^7^. Here we also confirmed that RRA significantly enhanced the DNA cleavage activities of original Cas12f-ge4.1 combination across 8 endogenous sites (Supplementary Fig. 2a). RRA was then introduced into d12f (hereafter dRRA) and significantly enhanced the A-to-G editing activities of N-/CL-d12fABE-8e (106 W)/-2-8e (106 W) but not internal DS128-/DS130-d12fABE-8e (106 W)/−2-8e (106 W) (hereafter d12fABEs with RRA mutation annotated as dRRAABEs) (Supplementary Fig. 2b-c).

Other TadA mutants, including original evolved TadA* and related mutants TadA* (V106W), TadA* (V82G), TadA* (K20A-R21A), TadA* (F148A), were then exploited for functional d12fABEs. Functional analysis revealed that the editing activities and scopes of TadA*-derived d12fABEs depended on TadA* mutants and fusing strategies. N-/DS128-/DS130-dRRAABE-TadA* mainly targeted A4 while CL-dRRAABE-TadA* displayed no detectable activity at 12f-sg89 site (Fig. 1d). N-/DS128-/DS130-dRRAABE-TadA* (82 G) targeted A4 similarly, with N-dRRAABE-TadA* (82 G) displaying the highest activity (28.7%) (Fig. 1d) while CL-dRRAABE-TadA* (82 G) mainly targeted A18 (38.3%) (Fig. 1d). Other TadA* mutants including TadA* (V106W), TadA* (K20A-R21A), TadA* (F148A) also generated functional d12fABEs with specific fusing strategies. For example, N-/DS130-dRRAABE-TadA* (106 W) targeted A4 while CL-dRRAABE-TadA* (20A21A) targeted A18 (Supplementary Fig. 3). The editing signatures of N-/CL-dRRAABE-TadA* (82 G) were further evaluated across 12 endogenous sites, and N-dRRAABE-TadA* (82 G) displayed editing window at A3-A4 (12.3%~24.1%) while CL-dRRAABE-TadA* (82 G) displayed editing window at A16-A17 (14.2%~15.2%), with modest editing activity at A18-A19 (5.3%~7.8%) (Fig. 1e).

We then performed transcriptome sequencing for RNA off-target analysis of representative d12fABEs and revealed that dRRAABEs-8e (106 W), dRRAABEs-2-8e (106 W) and dRRAABEs-TadA* (82 G) displayed obvious RNA off-target editing, inducing ~7~14 fold higher numbers of A-to-I edits as compared to control dRRA (Supplementary Fig. 4a). In consistent with previous studies, internal fusing strategy reduced RNA off-target editing, with DS128-/DS130-dRRAABE-8e (106 W) displaying the fewest numbers of A-to-I edits (Supplementary Fig. 4a). Additionally, Cas-dependent DNA off-target of representative d12fABEs were evaluated at 10 Cas-OFFinder predicted off-target loci corresponding to sgRNA 12f-sg72^6^, and detectable DNA off-target editing was observed at 4/10 off-target sites (Supplementary Fig. 4b), with CL-d12fABE-8e (106 W) displaying the lowest DNA off-target editing with 12~36 on-to-off-target ratios (Supplementary Fig. 4c). Cas-independent DNA off-target analysis with R-loop assay revealed that d12fABEs generally induced low Cas-independent off-target editing at all six sites (<12%), with CL-dRRAABE-TadA* (82 G) displaying the lowest (0.1%~0.32%) (Supplementary Fig. 5).

### Development of functional miniCBEs with high editing activities

**Fig. 2:**
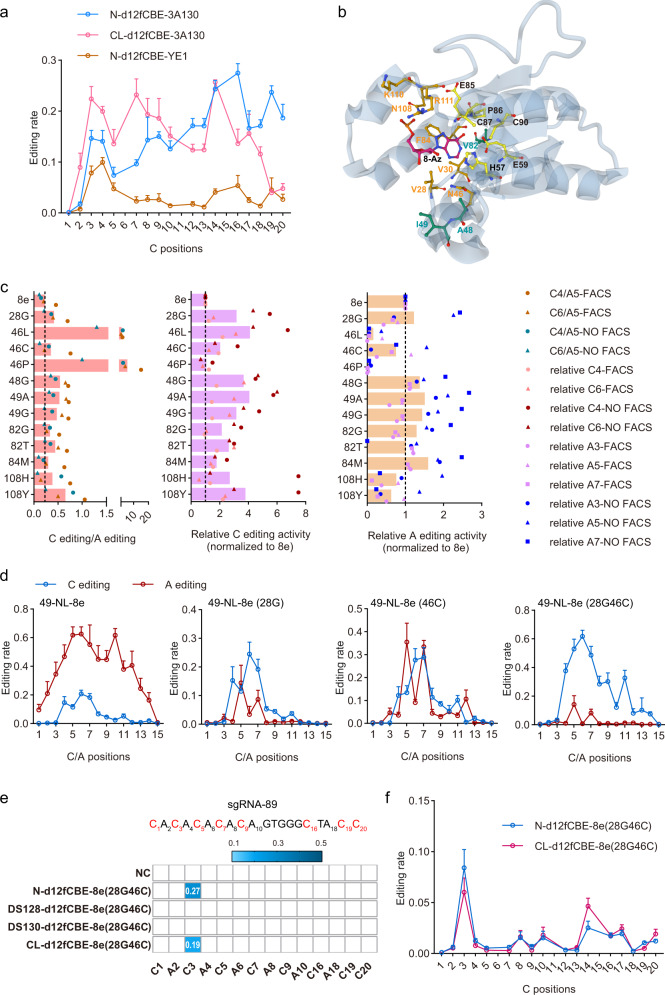
TadA engineering to generate functional d12fCBEs with high precision. **a** C-to-T editing signatures of N-d12fCBE-YE1, N-d12fCBE-3A130, and CL-d12fCBE-3A130 across 12 endogenous sites were shown in brown, blue, and pink lines, respectively. **b** Structure of TadA-8e (PDB code 6VPC), labeled with essential catalytic (black) and other residues (orange) within 5 Å of substrate analog 8-azanebularine (8-Az, black). Residues A48, I49, and V82, which were involved in pocket formation but out of 5 Å of 8-Az, were also labeled (cyan). **c** Representative single mutations enhancing C-to-T activity and preference. Bar graphs from left to right show ratios of C-to-T to A-to-G activity, relative C-to-T, and A-to-G efficiencies compared to 49-NL-8e, respectively. Editing signatures of 49-NL-8e variants were assessed at one representative endogenous site (sgRNA-1) in HEK293T cells. Two biologically independent screening experiments were performed, with FACS representing screening with flow-cytometry enrichment, and NO FACS representing screening without flow-cytometry enrichment. **d** Editing signatures of representative 49-NL-8e variants, including 49-NL-8e, 49-NL-8e (28 G), 49-NL-8e (46 C), and 49-NL-8e (28G46C) from left to right. Adenine editing (A editing) was shown in red lines and cytosine editing (C editing) was shown in blue lines. **e** Heatmap showing editing activities of d12fCBEs-8e (28G46C) against sgRNA 12f-sg89 in blue gradient color. **f** C-to-T editing signatures of N-d12fCBE-8e (28G46C) and CL-d12fCBE-8e (28G46C) across 12 endogenous sites in blue and violet line, respectively. NC, negative control. All data were collected from three independent experiments and presented as mean ± SEM. in histograms. Source data are provided as a Source Data file.

We further generated various potential miniCBEs by fusing rAPOBEC1 mutant (p. W90Y + p. R126E, hereafter YE1)^21,23,33^ or APOBEC3A mutant (p.Y130F, hereafter 3A130)^15^ to d12f as described above. Functional analysis revealed that N-/C-terminus fusing strategies generated functional miniCBEs (hereafter, d12fCBEs-YE1 and d12fCBEs-3A130, respectively) (Supplementary Fig. 6a, b). However, internal fusing strategies did not generate d12fCBEs with obvious editing activity. We further demonstrated that two additional cytidine deaminase mutants, APOBEC1 ancestor Anc689^18^ and APOBEC3A mutant (p.W98Y + p.W104A + p.Y130F, hereafter 3 A(YAF))^34^ also generated functional d12fCBEs by N-/C-terminus fusing strategies (Supplementary Fig. 6c, d). The editing signatures of N-d12fCBE-YE1, N-d12fCBE-3A130, and CL-d12fCBE-3A130 were evaluated across 12 endogenous sites. N-d12fCBE-YE1 displayed editing window at C3-C4 with relatively low editing activity (7.8%~9.9%) while N-d12fCBE-3A130 and CL-d12fCBE-3A130 displayed wide windows (C3-C18 and C3-C20, respectively) and higher editing activity (7.4%~27.5%, and 3.9%~25.7%, respectively) (Fig. 2a).

Consistently, RRA mutations also enhanced the editing activities of d12fCBEs (Supplementary Fig. 7a, b). In addition to N-/C-terminus fusing d12fCBEs, RRA also significantly enhanced the editing activities of internal fusing d12fCBEs-3A130 (5% to 15%) (Supplementary Fig. 7b). RRA mutations were also introduced into d12fCBEs-Anc689 and d12fCBEs-3A (YAF) and their editing signatures across 12 endogenous sites were evaluated. N-dRRACBE-Anc689 displayed complex editing window at C3-C5, C8-C12, C14-C16, and C19 (5.6%~17.8%) while CL-dRRACBE-Anc689 displayed editing window at C14-C16 and C19 (11.0%~15.8%) (Supplementary Fig. 8a). N-dRRACBE-3A (YAF) and CL-dRRACBE-3A (YAF) displayed wide windows (C3-C18 and C3-C20, respectively) with improved editing activities (9.0%~30.9% and 5.8%~28.0%, respectively) (Supplementary Fig. 8b). As these d12fCBEs could not enable efficient C-to-T editing and high precision simultaneously, alternative strategies are required for further improvement.

### TadA reprogramming to generate functional miniCBEs with high precision and minimized off-target effects

ABEs could catalyze C-to-T editing and specific mutations refined their substrate specificity^20,28,29^. Then TadA-8e was engineered to reprogram substrate specificity and adapted for optimized miniCBEs. ABE variant, with NLS-TadA-8e-linker domain inserted at residue 1249 of nCas9 (hereafter 49-NL-8e)^20^, was chosen for mutagenesis screening, as 49-NL-8e displayed obvious A-to-G and C-to-T activities (Supplementary Fig. 9). Generally, 10 residues involved in the pocket formation of TadA-8e, including V28, V30, N46, A48, A49, V82, F84, N108, K110 and R111 were selected for saturation mutagenesis^35^ (Fig. 2b). A total of 43 mutations at V28, N46, A48, I49, V82, F84 and N108 positions were identified as key mutations to enhance C-to-T activity and preference (Supplementary Fig. 10). Then 1~2 representative mutations at each position were selected for combinational screening (Fig. 2c), and 33 double mutation combinations displayed increased C-to-T activity and preference (Supplementary Fig. 11).

Then twelve 49-NL-8e variants were systematically analyzed across 12 endogenous sites. As compared to 49-NL-8e, single or double mutations significantly reprogramed editing windows and substrate specificity (Fig. 2d, Supplementary Fig. 12). For example, V28G and N46C single mutation reduced A-to-G activity, leading to comparable A-to-G and C-to-T activity, while V28G-N46C (28G46C) double mutation almost eliminated A-to-G activity, and displayed robust C-to-T activity (~60%) with editing window across C4-C11 (preferring C4-C7) (Fig. 2d). We then chose 8e (84M108Y), which displayed comparable C-to-T and A-to-G activities (Supplementary Fig. 12), and derived N-d12fBE-8e (84M108Y) displayed concurrent C-to-T and A-to-G activities with high precision, offering a promising miniACBEs for specific applications (Supplementary Fig. 13). As 49-NL-8e (28G46C) displayed the highest C-to-T activity with marginal A-to-G activity, d12fCBEs-8e (28G46C) were generated, with N-/CL-d12fCBE-8e (28G46C) displaying potent C-to-T activity at C3 (Fig. 2e). Further analysis across 12 endogenous sites also confirmed that N-/CL-d12fCBE-8e (28G46C) displayed precise editing window at C3 with modest C-to-T editing activity (8.4% and 6.0%, respectively) (Fig. 2f).

**Fig. 3:**
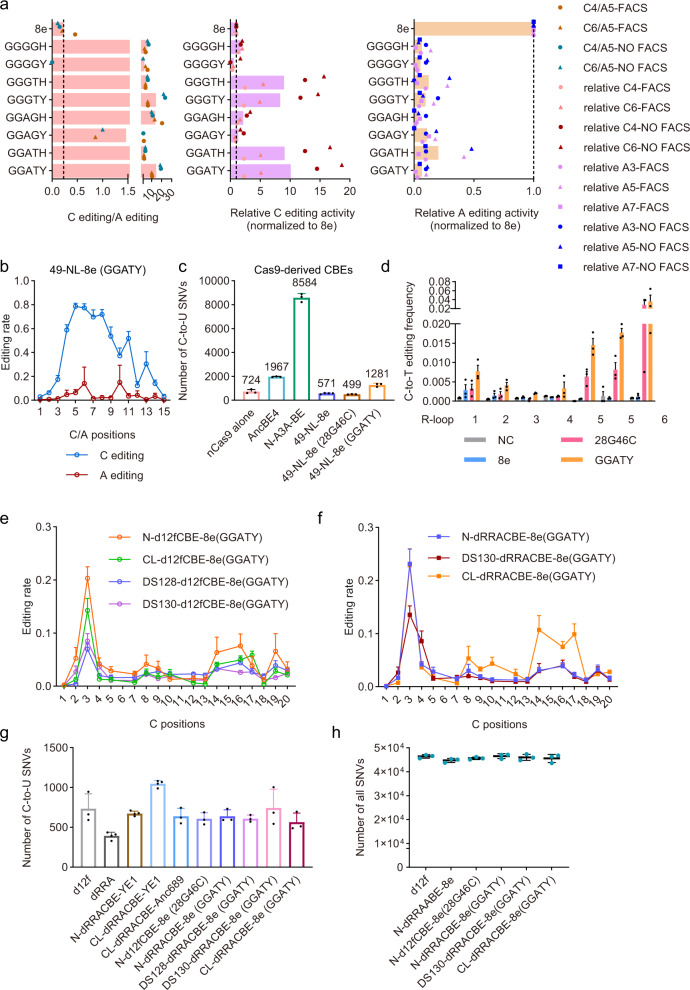
TadA reprogramming to generate d12fCBEs with improved properties. **a** Impact of quintuple mutations on C-to-T activity and preference. Bar graphs from left to right show ratios of C-to-T to A-to-G activity, relative C-to-T, and A-G efficiencies compared to 49-NL-8e, respectively. Editing signatures of 49-NL-8e variants were assessed at one representative endogenous site (sgRNA-1) in HEK293T cells. Two biologically independent screening experiments were performed, with FACS representing screening with flow-cytometry enrichment, and NO FACS representing screening without flow-cytometry enrichment. **b** Editing signatures of 49-NL-8e (GGATY). *n* = 3 biologically independent experiments. Adenine editing (A editing) was shown in red lines and cytosine editing (C editing) was shown in blue lines. **c** Number of cytosine-to-urine (C-to-U) RNA edits for classical CBEs AncBE4, N-A3A-BE and TadA-reprogrammed CBEs 49-NL-8e (28G46C), 49-NL-8e (GGATY). *n* = 3 or 4 (49-NL-8e) biologically independent experiments. **d** Cas9-independent off-target C-to-T conversion frequencies detected by R-loop assay at 6 R-loop sites with dSaCas9 and corresponding sgRNAs. Bars in the plots represent C positions showing the highest C-to-T activity at each R-loop site, with two C positions in R-loop 5 showing obvious C-to-T activity. *n* = 3 biologically independent experiments. **e**, **f** C-to-T editing signatures of TadA-reprogrammed d12fCBEs N-/CL-/DS128-/DS130-d12fCBE-8e (GGATY) (**e**) in orange, green, blue, and purple line, respectively; and N-/CL-/DS128-/DS130-dRRACBE-8e (GGATY) (**f**) in blue, red and orange line respectively, across 12 endogenous sites. *n* = 3 biologically independent experiments. **g** Number of C-to-U RNA edits for representative miniCBEs. *n* = 3 biologically independent experiments. **h** Total number of all SNVs relative to the parent sample detected by WGS. *n* = 3 biologically independent experiments. NC negative control. Data were presented as mean ± SEM. in histograms. Source data are provided as a Source Data file.

As d12fCBEs-8e (28G46C) displayed comparable C-to-T editing activity to d12fCBEs-YE1 but lower than d12fCBEs-3A130, combinations of >2 mutations were screened for further improvement. In addition to mutation combinations including N46L/P/C, double mutations V28G-N108H/C/Y and I49A-N108Y also displayed minimized A-to-G activity (Supplementary Fig. 14), indicating that substrate specificity could be reprogrammed via mutation combinations without N46L/P/C. Then all possible triple, quadruple and quintuple mutation combinations of V28G, A48G, I49A/G, V82G/T, and N108H/Y were screened. Overall, 13/38 triple (Supplementary Fig. 15), 21/26 quadruple (Supplementary Fig. 16), and all 8 quintuple mutations (Fig. 3a) displayed enhanced C-to-T activity and reduced A-to-G activity at all three A3, A5, A7 sites. Out of these mutations, V28G-A48G-I49A-V82T-N108Y (hereafter 8e (GGATY)) displayed the highest C-to-T and minimized A-to-G activity. Comprehensive editing signature analysis revealed that 49-NL-8e (GGATY) displayed higher C-to-T activity (~80%) with a wider window across C4-C11 (Fig. 3b) than 49-NL-8e (28G46C) (Fig. 2d).

Additionally, transcriptome analysis revealed that C-to-U RNA off-target edits of 49-NL-8e (28G46C) and −8e (GGATY) were largely eliminated and comparable to nCas9 but significantly fewer than classical CBE N-A3A-BE (Fig. 3c). Additionally, A-to-I RNA off-target edits of 49-NL-8e (28G46C) and −8e (GGATY) was also eliminated (Supplementary Fig. 17).

R-loop assay was also performed to evaluate Cas9-independent DNA off-target effects, the major safety concerns of CBEs^19,21^. 49-NL-8e displayed comparable A-to-G editing activity (0.25%~5.1%) to previous studies^32^, and selected mutations led to minimized A-to-G conversion activity (<1.5%) (Supplementary Fig. 18a). As expected, mutations for substrate specificity reprogramming increased C-to-T conversion activity (0~4% for 28G46C, 0.1%~6.4% for GGATY), but comparable to reported CBEs with eliminated Cas9-independent DNA off-target effects^21^ (Supplementary Fig. 18b, Fig. 3d). Then TadA-reprogrammed d12fCBEs-8e (GGATY) were generated and N-/CL-/DS130-d12fCBE-8e (GGATY) displayed obvious C-to-T activity with high precision (C3) (Supplementary Fig. 19a). RRA mutations enhanced C-to-T activity of d12fCBEs-8e (GGATY) by 20%~30% without compromising editing precision (Supplementary Fig. 19b, c). Editing signatures of TadA-reprogrammed d12fCBEs-8e (GGATY) with/without RRA mutations were further evaluated across 12 endogenous sites. It was shown that all d12fCBEs-8e (GGATY) variants displayed C-to-T editing activities with a single-base resolution editing window at C3, with CL-dRRACBE-8e (GGATY) displaying modest editing activity at C14-C17 (Fig. 3e, f). The editing activities of N-/CL-/DS128-/DS130-d12fCBE-8e (GGATY) at C3 were 20.3%±8.4%, 14.3%±8.7%, 7.0%±4.0% and 8.5%±5.5% respectively while N-/CL-/130-dRRACBE-8e (GGATY) at C3 were 23.1%±10.8%, 22.9%±12.0% and 13.6%±6.4% respectively (Fig. 3e, f).

Additionally, we analyzed the context preference of TadA-reprogrammed d12fCBEs. N-d12fCBE-8e (28G46C) preferred CA > CT > CC/CG & AC/CC/GC > TC (underline for target) while CL-d12fCBE-8e (28G46C) preferred CA > CT > CC/CG & TC > CC > AC/GC (Supplementary Fig. 20a). Nevertheless, N-dRRACBE-8e (GGATY) slight preferred CA (*P* = 0.057), DS130-dRRACBE-8e (GGATY) preferred CA/CC/CT > CG & TC > GC/CC/AC while CL-dRRACBE-8e (GGATY) preferred CA/CT > CC > CG & CC > AC/GC/TC (Supplementary Fig. 20b). As CBEs usually induced C-to-G/A mutations in addition to C-to-T mutation, we also analyzed the product purity and revealed that an average of >95% edited reads induced by d12fCBEs were C-to-T mutations (Supplementary Fig. 20c).

We further performed transcriptome sequencing for RNA off-target analysis and revealed that cytidine deaminase-derived d12fCBEs-YE1/Anc689 and TadA-reprogrammed d12fCBEs-8e (28G46C)/8e (GGATY) displayed minimal C-to-U and A-to-I RNA off-target edits (Fig. 3g, Supplementary Fig. 21). Cas-dependent DNA off-target analysis was performed at 21 Cas-OFFinder predicted off-target loci corresponding to three on-target loci^6^. It was revealed that DS130-dRRACBE-8e (GGATY) displayed 1.5~810 on-to-off-target ratios across all 8 off-target sites of 12f-sg6 (Supplementary Fig. 22) and 1.4~145 on-to-off-target ratios across all 9 off-target sites of 12f-sg72 (Supplementary Fig. 23). For other d12fCBEs, on-to-off-target ratios at ≥1 off-target sites were below 1, indicating higher off-target editing than on-target editing (Supplementary Fig. 22-23). For all 4 off-target sites of 12f-sgEMX, no detectable off-target DNA edits were induced (Supplementary Fig. 24).

Next, we also performed R-loop assay to evaluate Cas-independent DNA off-target editing for representative d12fCBEs. In consistent with classical CBEs^21^, d12fCBEs containing 3A130, 3 A (YAF) or Anc689 induced robust C-to-T edits (up to 41% for 3A130, 35% for 3 A (YAF) and 36% for Anc689) while YE1, an optimized mutant with minimized Cas-independent DNA off-target editing, induced just up to 10% C-to-T edits across all six sites (Supplementary Fig. 25). TadA-reprogrammed d12fCBEs induced comparable C-to-T edits to YE1(up to 12% for 8e (28G46C) and 10% for 8e (GGATY)) (Supplementary Fig. 25). Whole-genome sequencing (WGS) analysis also confirmed that total single-nucleotide variants (SNVs) were similar between d12fCBEs-8e (GGATY)- and d12f-treated HEK293T cells (Fig. 3h). Taken together, TadA-reprogrammed d12fCBEs represented optimized miniCBE tools with potent editing activity, high precision and minimized DNA/RNA off-target effects.

### Precise rescue and induction of pathogenic mutations in vitro and in vivo

**Fig. 4:**
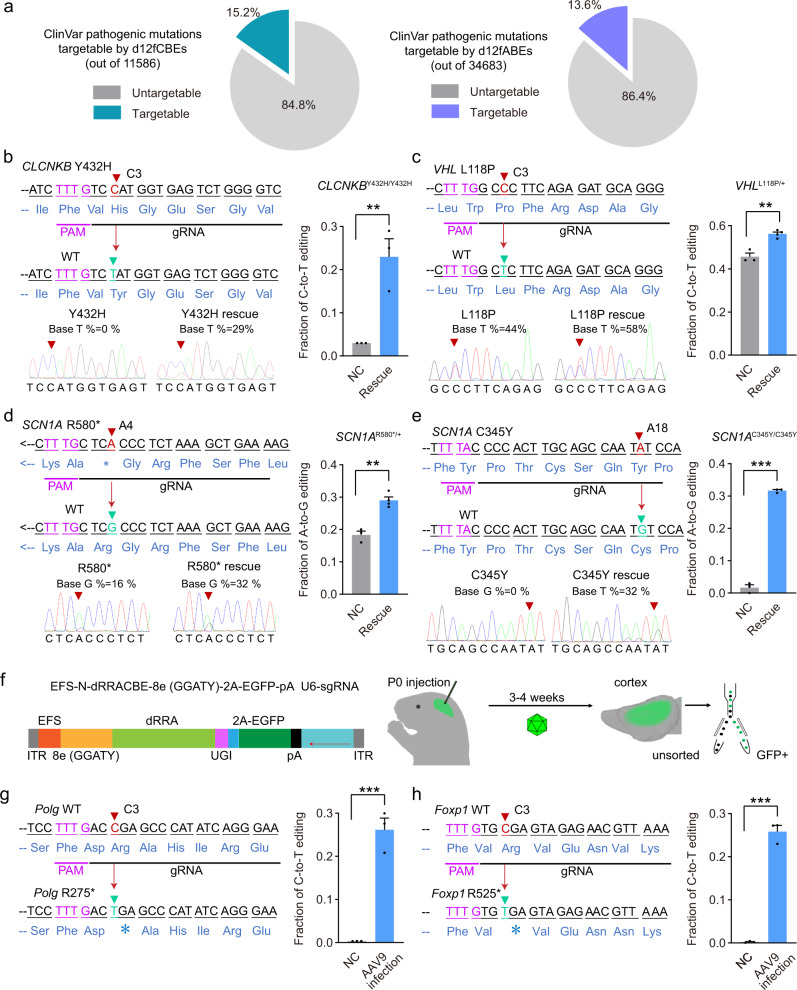
Application potentials of TadA-reprogrammed d12fCBEs and d12fABEs with improved properties. **a** Frequencies of genetic mutations in ClinVar that could be corrected by d12fCBEs (cyan) and d12fABEs (blue) in principle. **b**, **c** Design of sgRNA targeting to the mutation site in *CLCNKB* gene (**b**) or *VHL* gene (**c**) and rescue with TadA-reprogrammed N-dRRACBE-8e (GGATY). **d** Design of sgRNA targeting to the mutation site in *SCN1A* gene and rescue with N-dRRAABE-TadA* (82 G). **e** Design of sgRNA targeting to the mutation site in *SCN1A* gene and rescue with CL-dRRAABE-TadA* (82 G). Representative sanger sequencing results were shown, with histogram showing the correction efficiency. **f** Schematic diagram showing the AAV-delivered d12fCBEs and workflow for inducing pathogenic mutations in mouse brain. Generally, P0 intraventricular injection was performed, and GFP-positive cortical neurons were collected by flow cytometry 3–4 weeks after injection for high-throughput sequencing analysis. **g**–**h** Design of sgRNAs expected to install pathogenic mutation in *Polg* gene (**g**) or *Foxp1* gene (**h**), with a histogram showing the editing efficiency. *n* = 3 or 4 (*VHL* rescue and *SCN1A* R580 rescue) biologically independent experiments, and presented as mean ± SEM. in histograms. **p < 0.01, ***p < 0.001 with two-tailed unpaired *t* test. Exact *P* values for **b**–**e**, **g**–**h** were as follows, *P* = 0.00862, 0.00166, 0.00123, 5.81e-06, 0.000721, and 5.61e-05, respectively. PAM sequence was labeled as violet; target base was labeled as red while resulting in base was labeled as green. NC negative control. Source data are provided as a Source Data file.

Ca12f-derived miniABEs and miniCBEs are promising therapeutic tools for genetic diseases. In consideration of the editing windows of all functional miniABEs and miniCBEs described here (with miniABEs targeting A2-A18 and miniCBEs targeting C3-C20), 15.2% of A-to-G and 13.6% of C-to-T variants in ClinVar located in A2-A18 & C3-C20 (TTTR PAM, R counted as 0, targetable by d12fBEs in this study) and therefore could be corrected by d12fCBEs and d12fABEs theoretically^36^ (Fig. 4a). Among d12fCBEs-targetable pathogenic mutations, about 4.7% could be precisely corrected by TadA-reprogrammed d12fCBEs (about 0.64% pathogenic mutations in ClinVar database, Supplementary Fig. 26a). Among d12fABE-targetable mutations, about 7.0% could be precisely corrected by N-dRRAABE-TadA* (82 G) while 6.1% could be precisely corrected by CL-dRRAABE-TadA* (82 G) (~1.1% and 0.93% pathogenic mutations in ClinVar database respectively, Supplementary Fig. 26b, c).

In proof-of-concept applications, we established cell models carrying particular pathogenic mutations suitable for therapeutic treatment with d12fBEs. In cells carrying *CLCNKB*^Y432H/ Y432H^ or *VHL*^L118P/+^ mutations, TadA-reprogrammed N-dRRACBE-8e (GGATY) corrected corresponding pathogenic mutations efficiently (0.3% to 23% for *CLCNKB* and 44% to 56% for *VHL*) (Fig. 4b, c). Additionally, in another two cell models, N-dRRAABE-TadA* (82 G) corrected *SCN1A* R580* mutation efficiently (17.5% to 29%, Fig. 4d) and CL-dRRAABE-TadA* (82 G) corrected *SCN1A* C345Y mutation efficiently (0.2% to 32%, Fig. 4e).

Additionally, d12fCBEs were promising tools to model pathogenic mutations in vitro and in vivo for functional analysis. We examined the applicability of representative miniCBEs to induce 11 different pathogenic mutations at 10 sites in HEK293T cells. Cytidine deaminase-derived d12fCBEs generally displayed potent C-to-T editing activity to induce desired pathogenic mutations and bystander mutations simultaneously (Supplementary Fig. 27). N-dRRACBE-YE1 and CL-dRRACBE-Anc689 displayed improved precision but only induced 3/11 pathogenic mutations at 3/10 sites precisely (Supplementary Fig. 27). On the other side, TadA-reprogrammed d12fCBEs displayed potent C-to-T editing activity and high precision simultaneously, with N-dRRACBE-8e (GGATY) inducing 11/11 desired pathogenic mutations at 10/10 sites with high precision (C3-C4) (Supplementary Fig. 27). We further examined the homogeneity of edited products and it was revealed that TadA-reprogrammed N-dRRACBE-8e (GGATY) induced 8/11 desired pathogenic mutations precisely at 8/10 sites with a high proportion of desired alleles (>50%) (Supplementary Fig. 28a). *IRF6* loci contained two nearby pathogenic mutations and could not be induced separately by N-dRRACBE-8e (GGATY) effectively. However, N-/CL-d12fCBE-8e (28G46C) induced IRF6 C_4_-to-T mutation with high efficiency (73.3% and 84.5%, respectively) (Supplementary Fig. 28b). Additionally, DS130-dRRACBE-8e (GGATY) induced IRF6 C_3_-to-T mutation with high efficiency (39.2%) (Supplementary Fig. 28b). Therefore, as compared to cytidine deaminase-derived d12fCBEs, TadA-reprogrammed d12fCBEs are more suitable to generate desired alleles without bystander mutations.

The performance of TadA-reprogrammed N-dRRACBE-8e (GGATY) in vivo was also examined. We prepared a recombinant AAV9 (rAAV9) system, reported to transduce the central nervous system efficiently^37^, to deliver N-dRRACBE-8e (GGATY) into mouse brain, together with sgRNAs expected to install specific C-to-T mutation in mouse *Polg* mimicking human c.823 C > T (R275*) or in mouse *Foxp1* mimicking human c.1573 C > T (R525*) pathogenic mutations (Fig. 4f, h). Following a single dose AAV injection, N-dRRACBE-8e (GGATY) resulted in obvious C-to-T editing in *Polg* loci or *Foxp1* loci in GFP-positive cortical neurons in mouse brain (an average of 26.1 ± 4.8% and 25.8 ± 2.5% editing frequency, respectively) (Fig. 4g-h), confirming the in vivo application potential of TadA-reprogrammed d12fCBEs. Additionally, as AAV delivery system would lead to continuous expression of base editors, the RNA off-target effects of in vivo editing were further examined for mice treated with a single dose AAV injection to induce C-to-T editing in *Foxp1* loci, and similar A-to-I and C-to-U RNA off-target edits were observed between control and AAV-miniCBE treated mice (Supplementary Fig. 29).

## Discussion

Base editors are powerful tools to enable targeted base substitutions precisely and efficiently. As most pathogenic genetic mutations are single-nucleotide polymorphisms (SNPs)^38–40^, base editors are considered promising gene therapy strategies with widespread application potentials. Nevertheless, classical base editors exceed the AAV capacity and thus could not be delivered efficiently with all-in-one AAV vector. Though split base editors have been generated to achieve efficient delivery by dual AVVs^41^, miniature base editors are urgently needed to enable all-in-one AAV delivery in vivo for therapeutic applications. Actually, previous studies have only generated functional miniABEs with Cas12f or its orthologue systems^7,11^, and no functional miniCBEs have been reported previously. Here we generate a series of potent miniABEs and miniCBEs with diversified editing signatures composed of various engineered deaminases and hypercompact CRISPR-Cas12f system, with the help of flow cytometry (Supplementary Fig. 30), thereby providing more choices of miniBEs.

Widespread applications of base editors are also hindered by editing precision and safety issues. Though various base editors have been generated, it is still a great challenge to achieve base substitutions at single-base resolution. Here we generate functional miniABEs and miniCBEs with significantly improved editing precision by TadA engineering and reprogramming. Cas12f-derived N-/CL-dRRAABEs-TadA* (82 G) enable potent A3-A4 editing and A16-A17 editing, respectively. What’s more, TadA-reprogrammed d12fCBEs enable potent C3 editing. We also demonstrate that TadA-reprogrammed d12fCBEs display minimized DNA/RNA off-target edits, in addition to potent editing activity and high precision.

We have demonstrated in another study published simultaneously that various TadA orthologs could be engineered to generate functional CBEs and ACBEs via single or double amino acid substitutions^42^. As ecTadA variant TadA-8e was reprogrammed to generate potent CBEs and miniCBEs with high precision in this study, engineering of TadA orthologs would provide more deaminase choices for the development of miniCBEs with diversified features to expand miniature base editing toolkits.

We also noticed that Un1Cas12f1 needs a strict TTTR PAM sequence for targeting, which would limit the targeting scopes of these base editors. Therefore, future work is required to develop engineered Un1Cas12f1 variants with expanded PAM sequences or examine the availability of additional hypercompact CRISPR systems with distinct PAM sequences for functional miniBEs. Furthermore, the editing scopes of miniBEs with high precision described in this study are still limited and further engineering to generate miniBE variants with shifted editing scopes is needed.

In summary, our study robustly expands the base editing toolkits that could be delivered with all-in-one AAV system and provides more therapeutic choices with improved editing activity for a wide range of genetic diseases. We also demonstrate that TadA reprogramming provides alternative strategy for base editing optimization. Actually, miniBEs described here would be adapted for widespread applications with further improvements, such as PAM-expanded Cas12f or IscB variants and engineered deaminases.

## Methods

### Ethical statement

Our research complies with all relevant ethical regulations, and animal experiments have been approved by and were in accordance with the guidelines of the Animal Committee of the Department of Laboratory Science, Fudan University, China.

### Plasmid construction

TadA-8e, Un1Cas12f (D326A, D510A), and ogeuIscB were synthesized commercially (GENEWIZ, from Azenta Life Sciences). Plasmids expressing base editors were derived from PX461 (Addgene #48140), with U6-sgRNA cassette deleted through BbsI+XbaI double digestion strategy. Plasmids expressing engineered ABEs with TadA-8e fusing inside nCas9 were constructed as described previously. In brief, docking sites containing SpeI-BamHI-XbaI multiple cloning sites at specific residues of nCas9 were generated via mutagenesis strategy (KOD-plus, Toyobo, Cat#: KOD-201). TadA-8e and NLS-TadA-8e-linker sequences were inserted into desired docking sites through BamHI-XbaI double digestion strategy (New England Biolabs). Plasmids expressing engineered miniBEs with deaminases fusing inside d12f were constructed similar as described above. Point mutation or mutation combinations were induced for plasmid 1249-NLS-TadA-8e-linker via mutagenesis strategy (KOD-plus, Toyobo, Cat#: KOD-201). Plasmids expressing dSaCas9-UGI-T2A-mCherry and U6-sgsaRNA were derived from PX602 (Addgene #61593), in which D10A and N580A were induced via mutagenesis strategy (KOD-plus, Toyobo, Cat#: KOD-201), and then UGI-T2A-mCherry cassette was inserted through BamHI-EcoRI double digestion strategy. Plasmids expressing sgRNAs were generated using U6-sgRNA-EF1alpha-UGI-T2A-mCherry as described previously. Additionally, mCherry was replaced with puromycin resistance gene to generate plasmid expressing sgRNAs for puromycin-based enrichment. Information for Cas12f and sgRNA scaffold ge4.1 was provided in Supplementary Data 1. The sgRNAs used in this study were listed in Supplementary Data 2. And all related primers for molecular cloning were purchased from GENEWIZ and listed in Supplementary Data 3.

### Cell culture and transfection procedure

HEK293T cells (GNHu17, Cell Bank of the Chinese Academy of Sciences, Shanghai, China) were cultured using DMEM (Sigma-Aldrich; Cat#: D5796) with 10% FBS (Gibco, Thermo Fisher Scientific; Cat#: 26010074) in 5% CO_2_ incubator (Heraeus, Thermo Fisher Scientific) at 37°C. HEK293T cells were plated into six-well or 48-well plates (Corning) 24 h before transfection. Then transfection was performed using Lipo293^TM^ (Beyotime Biotechnology, Shanghai, China; Cat#: C0521) according to the manufacturer’s instructions. Briefly, base editor-expressing plasmid and sgRNA-expressing plasmid (mole ratio 2:1) were mixed with 2.5~3 μl (48-well) or 8 μl (six-well) Lipo293^TM^ and incubated at room time for 10 minutes before adding into the wells. Cells were cultured for another 72 h and collected for base editing analysis. For puromycin-based enrichment and subsequent RNA-seq analysis, media was replaced with fresh media containing 2 μg/ml puromycin (Beyotime Biotechnology, Shanghai, China; Cat#: ST551-10mg) 24 h after transfection. Cells were collected within 72 h after transfection for RNA extraction to avoid the loss of RNA edits over time.

### Base editing analysis

Cells were collected directly (referred to as NO-FACS) or sorted by flow cytometry (referred to as FACS) to enrich GFP/mcherry double-positive cells (Moflo XDP, Beckman Coulter/BD FACSAria™ Fusion Flow Cytometers) with FlowJo v10. Collected cells were treated with DirectPCR reagent (Viagene Biotech, Ningbo, China; Cat#: 302-C) for subsequent PCR amplification using LA Taq (Takara, Dalian, China; Cat#: RR02MA). PCR products were analyzed using Sanger sequencing (BGI, Shenzhen, China) with EditR software or high-throughput sequencing (Shanghai Personalbio Technology, Shanghai, China) with CRISPResso2.

### Targeted amplicon sequencing

PCR primers for targeted amplicon sequencing were within 100 bp upstream or downstream of target sites. PCR products were verified on agarose gels and purified with Universal DNA purification kit (Tiangen Biotech, Beijing, China; Cat#: DP214). Amplicons of different target sites were mixed together for DNA library preparation (NEB). Deep sequencing was performed using Illumina NovaSeq platform at Shanghai Personalbio Technology. Experiments for amplicon sequencing were performed in biological triplicates.

### Analysis of Cas-independent DNA off-target effects

R-loop assay was used to evaluate the Cas9-independent DNA off-target effects as described previously. Briefly, base editor variants (expressing T2A-GFP cassette simultenously) were co-transfected with specific dSaCas9-UGI-T2A-mcherry-U6-sgsaRNA and cultured for another 72 hours. Double-positive cells were enriched by flow cytometry. PCR products of target sites were analyzed by high-throughput sequencing and CRISPResso2. The editing frequencies for adenine and cytosine were calculated and summarized separately. The sum of C-T frequencies for all cytosines or adenines within specific R-loop site was shown in the figure.

### Whole-genome sequencing and data analysis

HEK293T cells were transfected with Cas12f-derived tools and sgRNA-expressing plasmids. Here sgRNA-expressing plasmid containing puromycin resistance gene. Puromycin (Beyotime Biotechnology, Shanghai, China) was added into medium to a final of 2 μg/ml, and used for cell culture in 6-well plates every day for 4 days. Single-cell clone was picked up, expanded, and cultured for 3–4 weeks, Cell/Tissue Genomic DNA Kit (Tiangen Biotech, Beijing, China; Cat#: DP304) was used for cellular genome DNA purification and whole-genome sequencing was performed using MGIseq platform (Genewiz).

Paired-end sequencing reads were aligned to human genome build 38 (GRCh38/hg38) using BWA-MEM(v.0.7.17)^43^. Then bams were sorted, optical duplicates were marked and base-quality recalibration was performed with GATK (v.4.2.4) tools MarkIlluminaAdapters, SamToFastq, MergeBamAlignment, SortSam, MarkDuplicates, SetNmMdAndUqTags, BaseRecalibrator, and ApplyBQSR. A high-performance computing cluster was used for subsequent analyses. Google DeepVariant with default parameter^44^ was used for variant calling. Variant statistics were performed using custom shell scripts and Excel 2016.

### Animals

All animal breeding, care, and experiments have been approved by and were in accordance with the guidelines of the Animal Committee of the Department of Laboratory Science, Fudan University, China. The Postnatal day 0 (P0) C57BL/6 *Mus musculus* (Vital River Laboratories) was used in this study. Sex was not considered in this study design as *Foxp1* and *Polg* were autosomal genes and base editing was normally unaffected by sex differences. According to animal welfare requirements, all experimental mice were bred on the condition of a 12 h light/dark cycle (dark from 7 pm to 7 am while light from 7 a.m. to 7 p.m.) with food/water provided ad libitum. Additionally, pathogen-free (PF) unit under constant temperature (~22 °C), humidity (~55% RH), ventilation, and automatic circadian rhythm was maintained for experimental mice.

### P0 AAV intraventricular injection for in vivo base editing analysis

5 µl of AAV(1E + 13GC/ml, from PackGene Biotech) were mixed with 5% fast green into the injection syringe. Injection sites at 2/5 of the distance from the lambda suture to each eye were selected for AAV intraventricular injection. The syringe perpendicular was held to the surface of the skull with the needle inserted at the marked injection site to a depth of ~3 mm. Maximum volume of 1 µl virus was injected slowly into each ventricle until the dye-filled the ventricle.

### RNA-editing analysis by RNA-seq

RNA was extracted using Trizol reagent (Life Technologies, Thermo Fisher Scientific) for library preparation (NEB). The libraries were sequenced using Illumina Hiseq (PE 2 ◊150), at a depth of ~50 million reads per sample. We analyzed mRNA-seq data following the long-RNA-seq-pipeline of ENCODE Consortium. Reads were aligned to GRCh38 reference genome using annotation GRCh38.v96 via STAR (v2.4.2a) in 2-pass mode^45^. Gene counts were quantified using RSEM (v1.3.3)^46^. Variant calling from RNA-seq data was performed using Sentieon® genomics tools (v202010.02). Sequenced reads were first mapped to the reference genome (GRCh38) with STAR (v2.4.2a) in two-pass mode as described above. Picard (v2.23.6, http://broadinstitute.github.io/picard) was used to sort and mark duplicates of the mapped BAM files. After removing duplicates, reads were split at junctions into exon segments and reassigned the mapping qualities from STAR. Base-quality score recalibration (BQSR) was performed as the DNA-seq to remove experimental biases caused by the sequencing methodology. Then, variants were identified by MuTect2 tool (202010.02)^25,47^. To identify high-confidence variants, we performed hard-filtering using VariantFiltration tool of GATK with parameter --filter-expression ‘QUAL < 25||MQ < 20.0||QD < 2.0||FS > 30.0||DP<20’ to filter variants with base-quality score <25, mapping quality score <20, Fisher strand values >30.0, qual by depth values <2.0 or sequencing depth <20.

### Statistics and reproducibility

Prism 8.4.0 (GraphPad) was used for statistical analyses. Results in this study were presented as mean (heatmap) or mean ± standard error of mean (SEM) (bar plot). For two group comparisons, two-tailed unpaired *t* test was used. *P* < 0.05 was considered as statistical significance, with *P < 0.05, **P < 0.01, and ***P < 0.001. Exact *P* values were listed in corresponding figure legends or in Source Data. RNA-seq analysis was conducted using RSEM (v1.3.3), Sentieon genomics tools (v202010.02), STAR (v2.4.2a), Picard (v2.23.6, http://broadinstitute.github.io/picard), MuTect2 (202010.02) and VariantFiltration tool (gatk-4.1.4.0). Whole-genome sequencing analysis was conducted using BWA-MEM(v.0.7.17), GATK (v.4.2.4) tools MarkIlluminaAdapters, SamToFastq, MergeBamAlignment, SortSam, MarkDuplicates, SetNmMdAndUqTags, BaseRecalibrator and ApplyBQSR, Google DeepVariant and Excel 2016. *N* = 2–4 independent biological replicates were performed and listed in each figure. In this study, no statistical method was used to predetermine sample size, and no data were excluded from the analyses. The cell experiments were not randomized and the Investigators were not blinded to allocation during experiments and outcome assessment. Mice used for intraventricular injection were allocated to the control or AAV9-treated group randomly.

### Reporting summary

Further information on research design is available in the Nature Portfolio Reporting Summary linked to this article.

## Supplementary information

Supplementary Information

Description of Additional Supplementary Files

Peer Review File

Reporting Summary

Supplementary Data 1

Supplementary Data 2

Supplementary Data 3

Supplementary Movie 1

## Data Availability

The raw high-throughput sequencing data generated in this study have been deposited in the NCBI sequence Read Archive database under PRJNA918720. PDB: 6VPC used for structural analysis was available at. GRCh38 reference genome GRCh38.v96 used in this study is available at https://ftp.ensembl.org/pub/release-96/gtf/homo_sapiens/Homo_sapiens.GRCh38.96.gtf.gz. All plasmids described in this work are available, please contact the corresponding author T.L.C. (chengtianlin@fudan.edu.cn), and will be deposited to Addgene. Source data are provided with this paper.

## Source data

Source Data
