# Supplementary material for: TadA reprogramming to generate potent miniature base editors with high precision
Source: Nat Commun. 2023 Jan 26;14:413. doi: 10.1038/s41467-023-36004-2 (PMC9879996; doi:10.1038/s41467-023-36004-2)
Supplement: Supplementary file 1 — Supplementary Information [file 41467_2023_36004_MOESM1_ESM.pdf]

Supplementary Information

TadA reprogramming to generate potent miniature base editors with high precision

Zhang et al.

Supplementary Table 1 Location summary of potential internal docking sites for d12f-derived miniBEs.

Name	Start/end site for deaminase domain insertion（d12f）	Sequence around insertion site
N-d12f	1/NLS	M/PKKKR
DS61-d12f	61/62	AAYCT/TQVER
DS128-d12f	128/129	IFIKG/KGIAN
DS130-d12f	130/131	IKGKG/IANAS
DS211-d12f	211/212	ISNHN/SDFII
DS226-d12f	226/227	RWQVK/KEIDK
DS229-d12f	229/230	VKKEI/DKYRP
DS237-d12f	237/238	RPWEK/FDFEQ
DS245-d12f	245/246	EQVQK/SPKPI
DS283-d12f	283/284	MNGDY/QTSYI
DS226-238-d12f	226/238	RWQVK/FDFEQ
DS226-246-d12f	226/246	RWQVK/SPKPI
C-d12f	529- . Nucleoplasmin NLS/stop	AKKKK/*
CL-d12f	529- . Nucleoplasmin NLS-linker/stop	TSGSG/*

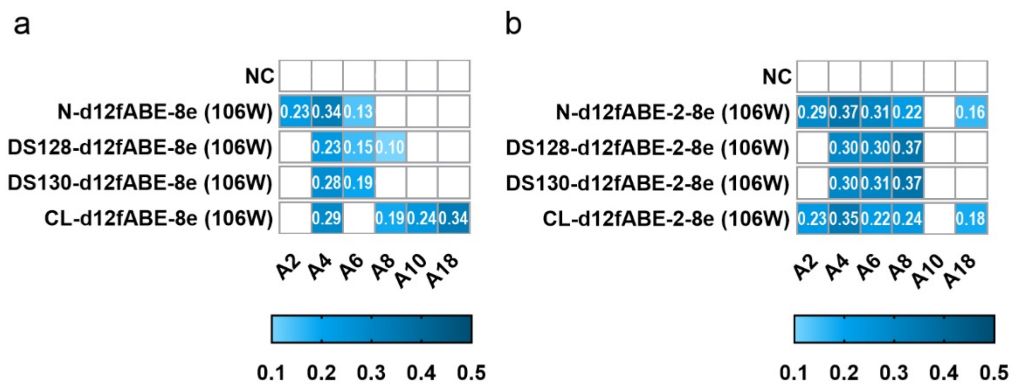

Supplementary Figure 1. Editing activities of d12fABEs-8e (V106W) and d12fABEs-2-8e (V106W). More d12fABEs were generated with 8e (V106W) and 2-8e (V106W)) by N-, C-terminus and internal 128/130-fusing strategies. Editing activities were evaluated against sgRNA 12f-sg89. Heatmaps from left to right showing A-to-G editing frequencies of d12fABEs-8e (V106W) (**a**) and d12fABEs-2-8e (V106W) (**b**) in blue gradient color. n=3 biologically independent experiments. NC, negative control. Source data are provided as a Source Data file.

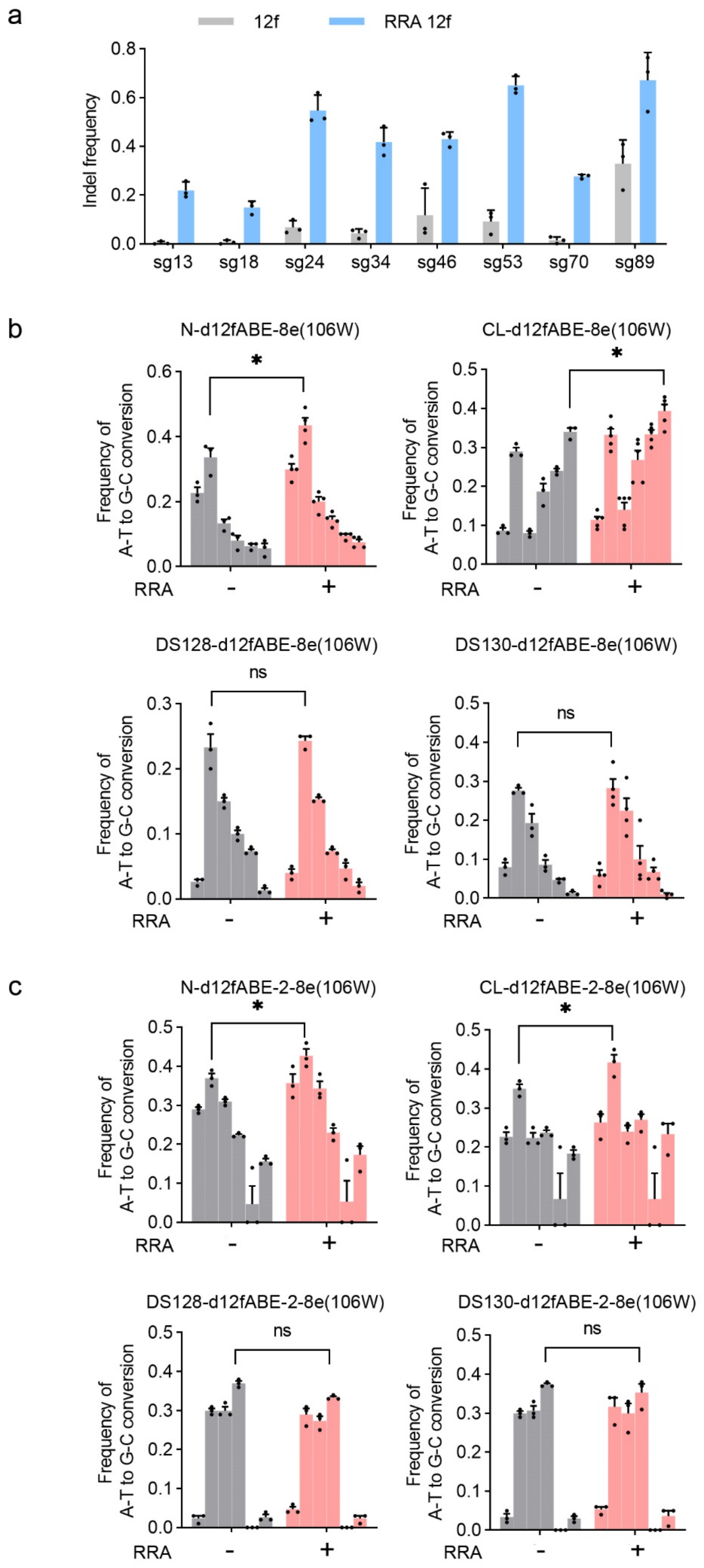
a
12f
RRA 12f
Indel frequency
0.0
0.2
0.4
0.6
0.8
sg13
sg18
sg24
sg34
sg46
sg53
sg70
sg89
b
N-d12fABE-8e(106W)
CL-d12fABE-8e(106W)
DS128-d12fABE-8e(106W)
DS130-d12fABE-8e(106W)
Frequency of A-T to G-C conversion
*
ns
RRA
-
+
c
N-d12fABE-2-8e(106W)
CL-d12fABE-2-8e(106W)
DS128-d12fABE-2-8e(106W)
DS130-d12fABE-2-8e(106W)

Supplementary Figure 2. Enhancement of editing activities for Cas12f and derived d12fABEs by RRA mutations. a, Indel frequencies of Cas12f (gray) and RRA Cas12f mutants (blue) coupled with sgRNA ge4.1 at 8 endogenous sites. n=3 biologically independent experiments. **b-c,** Histogram showing A-to-G editing comparison of d12fABEs-8e (106W) (**b**) and d12fABEs-8e (106W) (**c**) with or without RRNA mutations against sgRNA 12f-sg89. Data was collected from at least three independent experiments and presented as mean ± SEM. in histograms. *p<0.05 with two-tailed unpaired t-test and ns means no significance. Exact P values for b and c were as follows: b, P=0.043, 0.0394, 0.663, 0.848; c, P=0.0317, 0.046, 0.163, 0.822. n=3 biologically independent experiments. Source data are provided as a Source Data file.

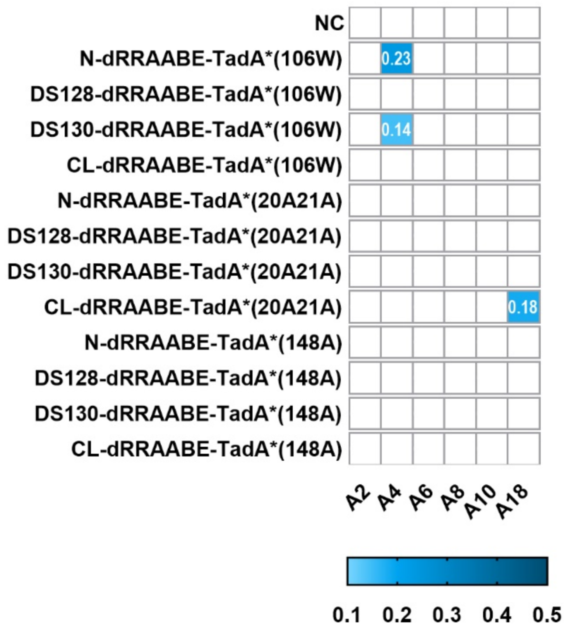

Supplementary Figure 3. Editing activities of d12fABEs-TadA* variants. d12fABEs N-/DS128-/DS130-/CL-dRRAABE-TadA* (106W), N-/DS128-/DS130-/CL-dRRAABE-TadA* (20A21A) and N-/DS128-/DS130-/CL-dRRAABE-TadA* (148A) were generated by different fusing strategies and editing activities were evaluated against sgRNA 12f-sg89. Heatmaps showing A-to-G editing frequencies of corresponding d12fABEs- TadA* variants in blue gradient color. Data was collected from at least three independent experiments. NC, negative control. Source data are provided as a Source Data file.

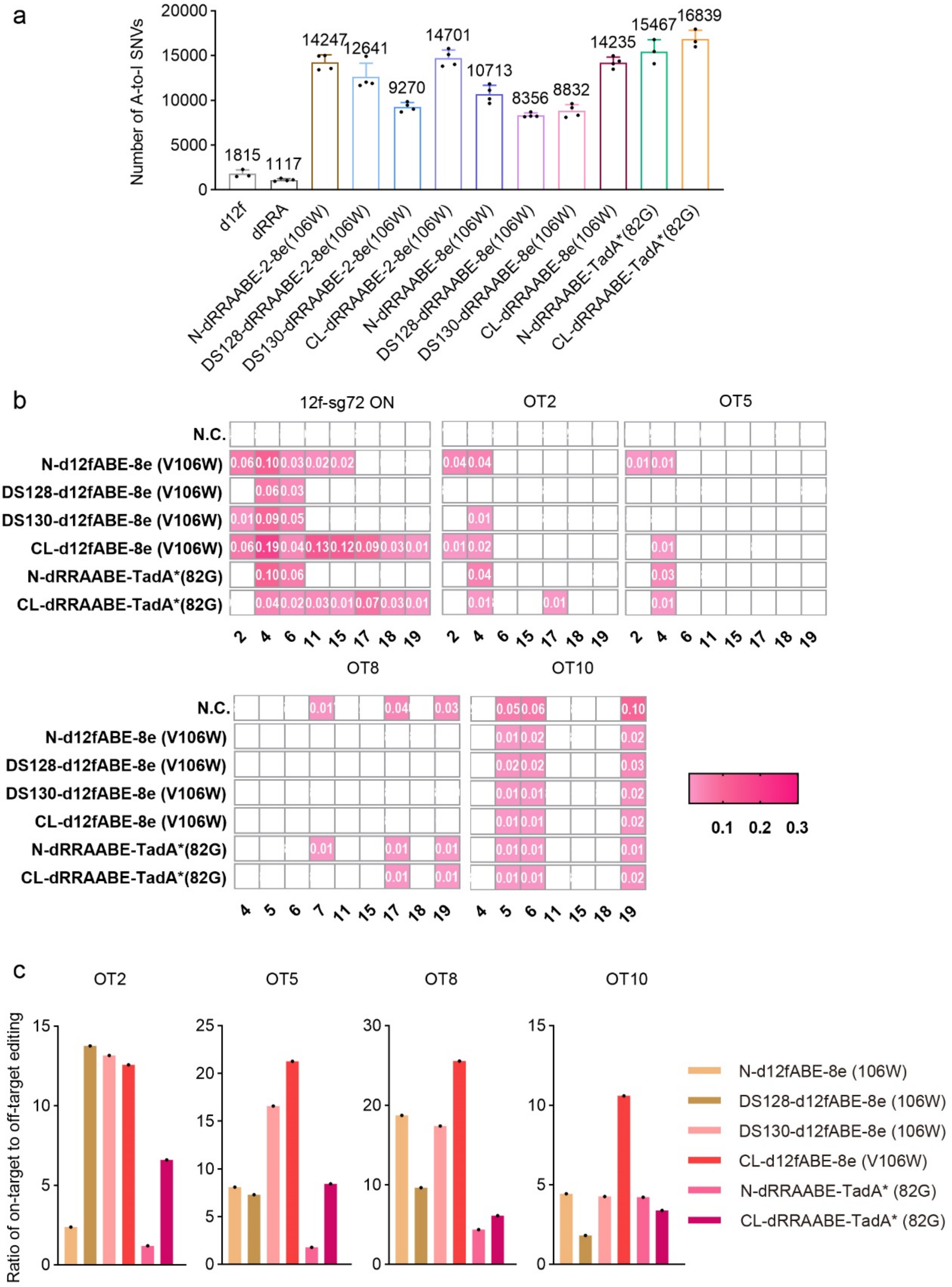

Supplementary Figure 4. RNA and DNA off-target effects of representative d12fABEs. a, Transcriptome analysis with MuTect2 software revealed the number of A-to-I edits in HEK293T cells transfected with corresponding d12fABEs and 12f-sg89.

Data here are presented as mean with SEM. n=3 (d12f, N-dRRAABE-TadA*(82G), CL-dRRAABE-TadA*(82G)) or 4 biologically independent experiments. **b,** Heatmaps showing the A-to-G editing activities of representative d12fABEs N-/DS128-/DS130-/CL-d12fABE-8e (106W) and N-/CL-d12fABE-TadA* (82G) at 12f-sg72 site and the top four potential off-target sites in pink gradient color. n=3 biologically independent experiments. **c,** On-to-off-target ratios of the top four potential off-target sites were quantified as follows: the highest on-target editing frequency/the highest off-target editing frequency. n=3 biologically independent experiments, and presented as mean ± SEM. in histograms. NC, negative control. Source data are provided as a Source Data file.

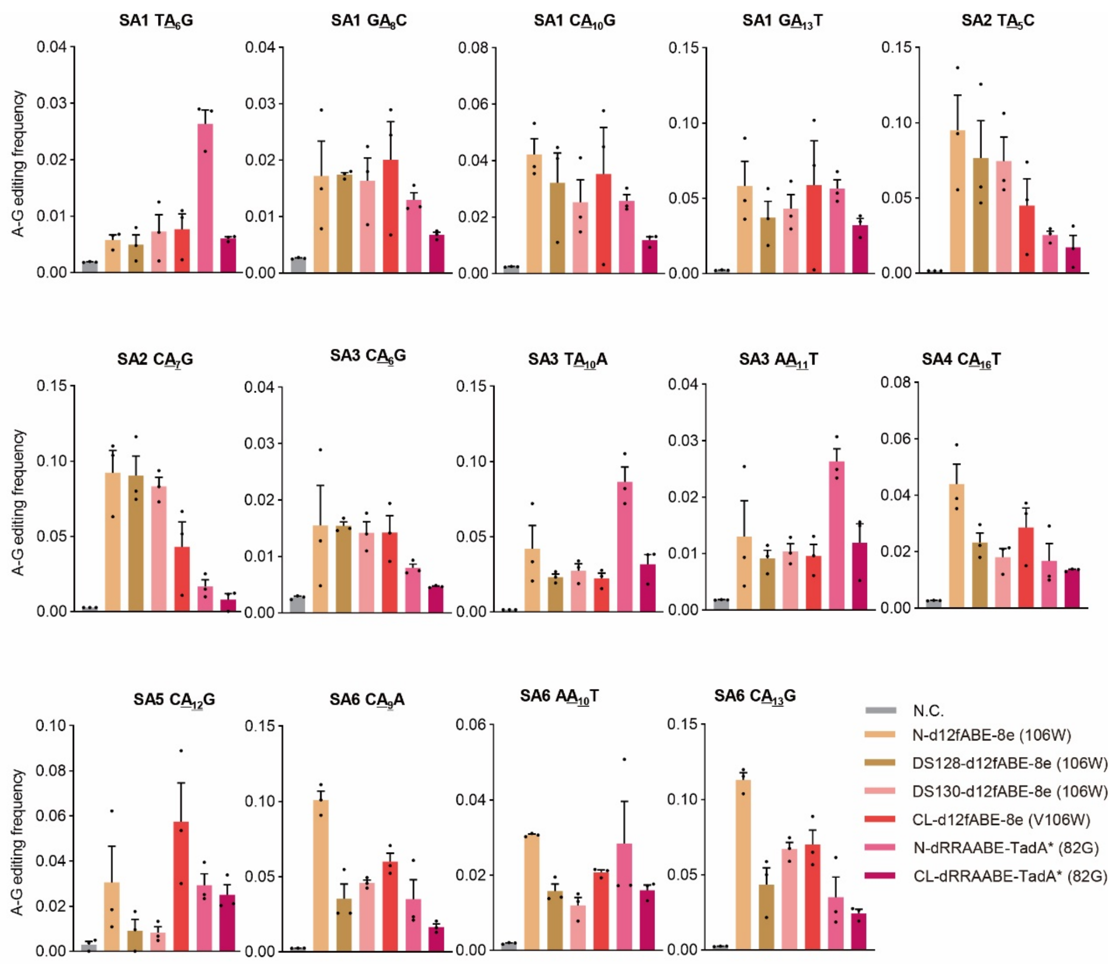

Supplementary Figure 5. Cas-independent DNA off-target effects of representative d12fABEs. Cas-independent off-target A-to-G editing frequency of representative d12fABEs N-/DS128-/DS130-/CL-d12fABE-8e (106W) and N-/CL-d12fABE-TadA* (82G) were analyzed by R-loop assay. Data was collected from three independent experiments and presented as mean ± SEM. in histograms. NC, negative control. Source data are provided as a Source Data file.

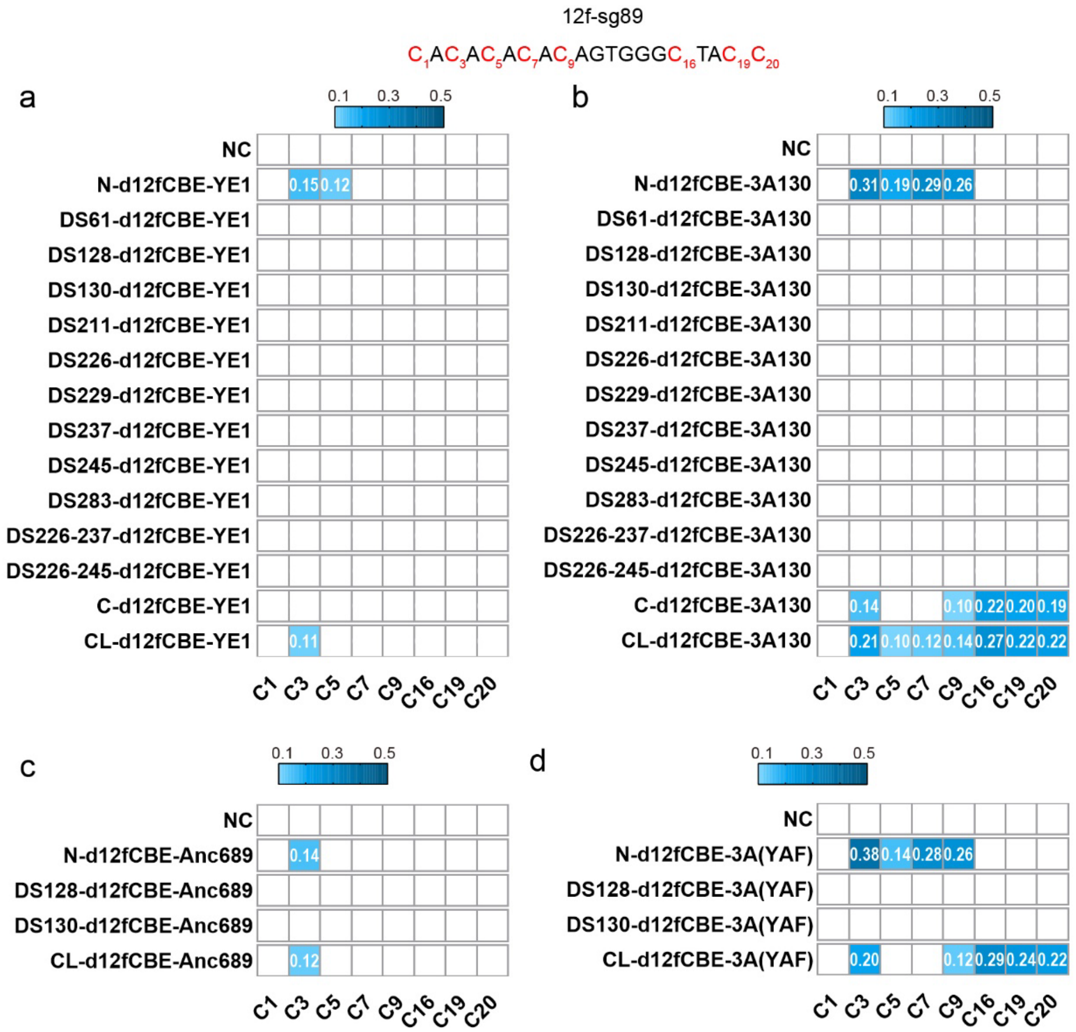

Supplementary Figure 6. Editing activities of potential d12fCBEs. a-b, Heatmaps from left to right showing C-to-T editing frequencies of d12fCBEs-YE1 (**a**) and d12fCBEs-3A130 (**b**) in blue gradient color. Various potential d12fCBEs were generated by fusing rAPOBEC1 mutant (p. W90Y+p. R126E, hereafter YE1) and APOBEC3A mutant (p.Y130F, hereafter 3A130) to N-/C-terminus or internal sites of d12f. Editing activities were evaluated against sgRNA12f-sg89. **c-d,** Heatmaps from left to right showing C-to-T editing frequencies of d12fCBEs-Anc689 (**c**) and d12fCBEs-3A (YAF) (**d**) in blue gradient color. Potential d12fCBEs-Anc689 and d12fCBEs-3A (YAF) were generated by N-, C- and internal fusing strategies in blue gradient color. Editing activities were evaluated against sgRNA 12f-sg89. Data was

collected from at least independent experiments. NC, negative control. Source data are provided as a Source Data file.

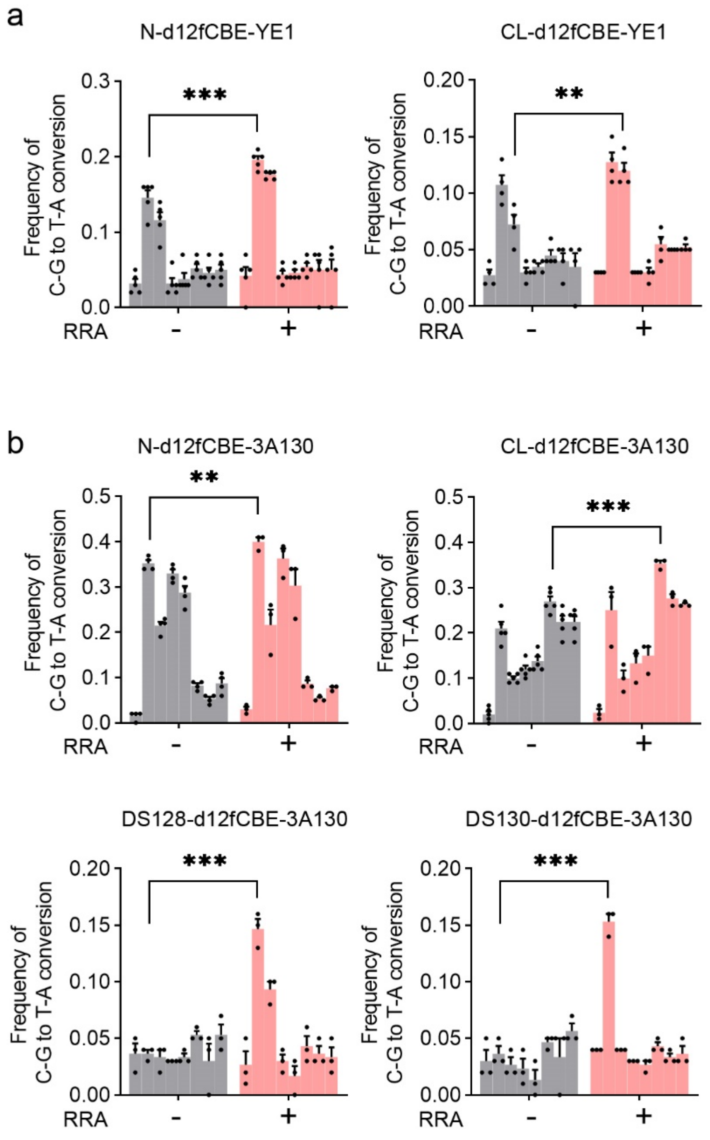

Supplementary Figure 7. Enhancement of editing activities for d12fCBEs by RRA mutations. a-b, Histogram showing A-to-G editing comparison of d12fCBEs-YE1 (**a**) and d12fABEs-3A130 (**b**) with or without RRNA mutations against sgRNA 12f-sg89. Data was collected from at least three independent experiments and presented as mean ± SEM. in histograms. ***p*<0.01, ****p*<0.001 with two-tailed unpaired t-test. n=3 biologically independent experiments. Exact *P* values were as follows, a, *P*=0.000471, 0.00518; b, *P*=0.00468,0.000447, 0.000309,0.000245. Source data are provided as a Source Data file.

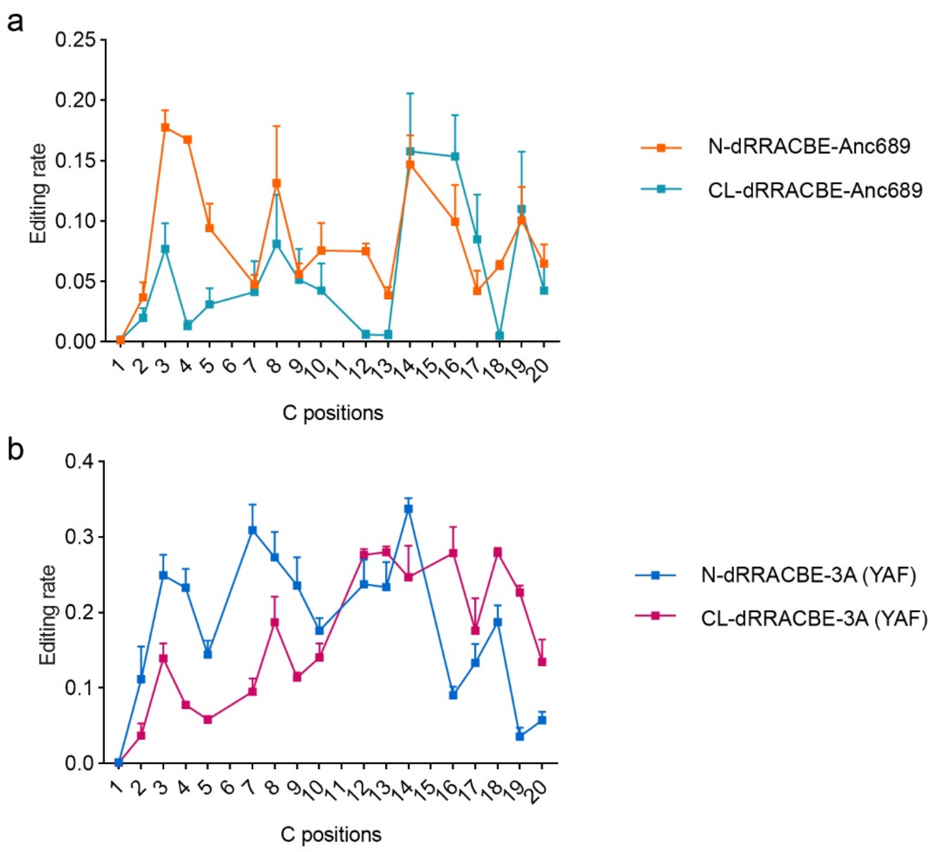

Supplementary Figure 8. Editing signatures of representative d12fCBEs-Anc689 and d12fCBEs-3A (YAF) with RRA mutations. A-to-G editing frequencies at each adenine position across 12 endogenous sites were quantified and summarized for N-/CL-dRRACBE-Anc689 (**a**) in orange and green line, repectively and N-/CL-dRRACBE-3A (YAF) (**b**) in blue and violet line, respectvely. Data was collected from three independent experiments and presented as mean ± SEM. in histograms. Source data are provided as a Source Data file.

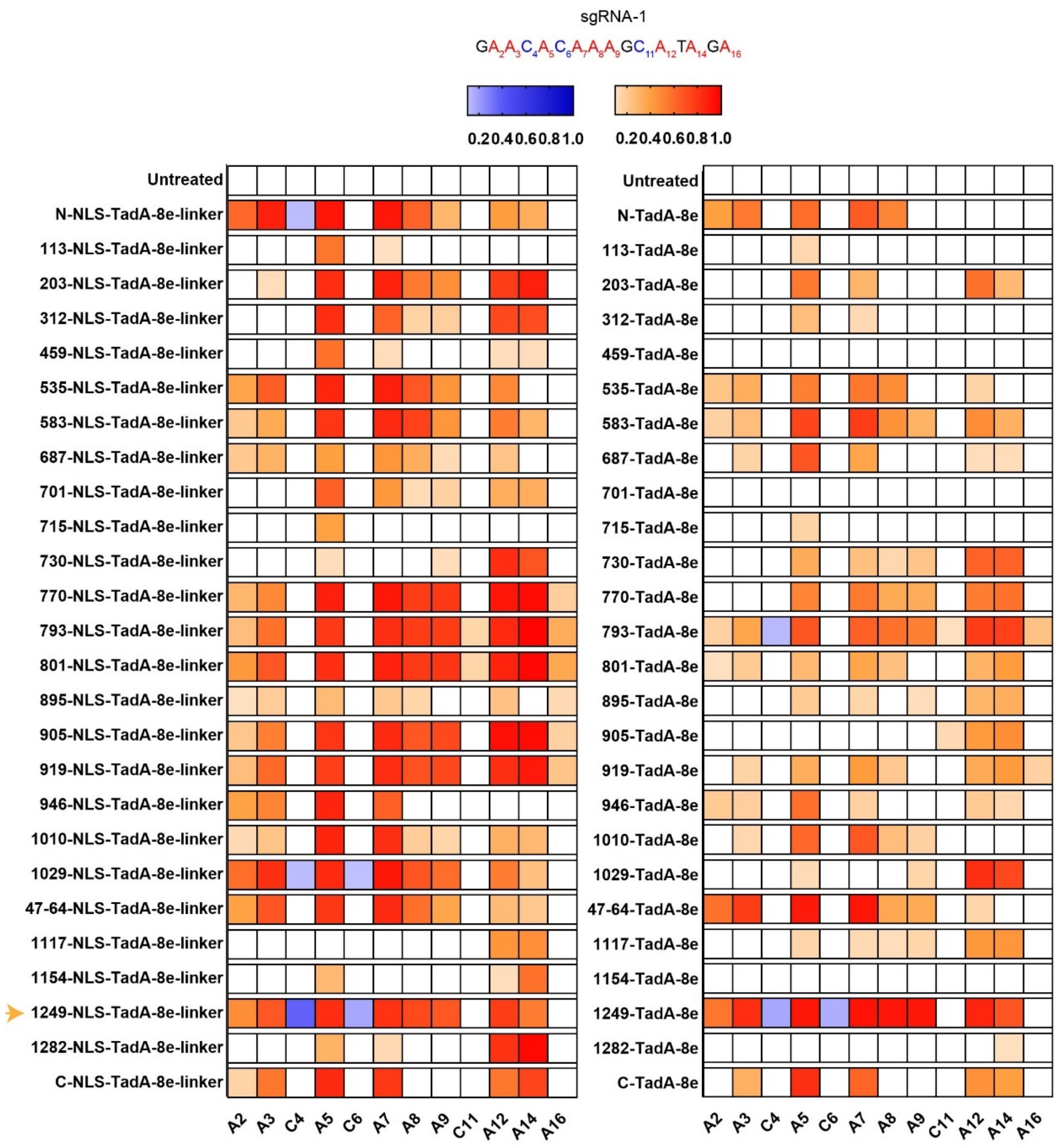

Supplementary Figure 9. Editing signatures of engineered ABEs generated by combination of domain insertion with two forms of TadA-8e. The editing frequencies are shown in heatmap format, with cytosine editing efficiencies (C-T editing) shown in blue and adenine editing efficiencies (A-G editing) in orange-red double gradient color. Engineered ABEs were generated by combination of domain insertion with NLS-TadA-8e-linker (left) or TadA-8e (right). Yellow arrowhead refers to ABE variant displaying the highest C-to-T activity. Source data are provided as a Source Data file.

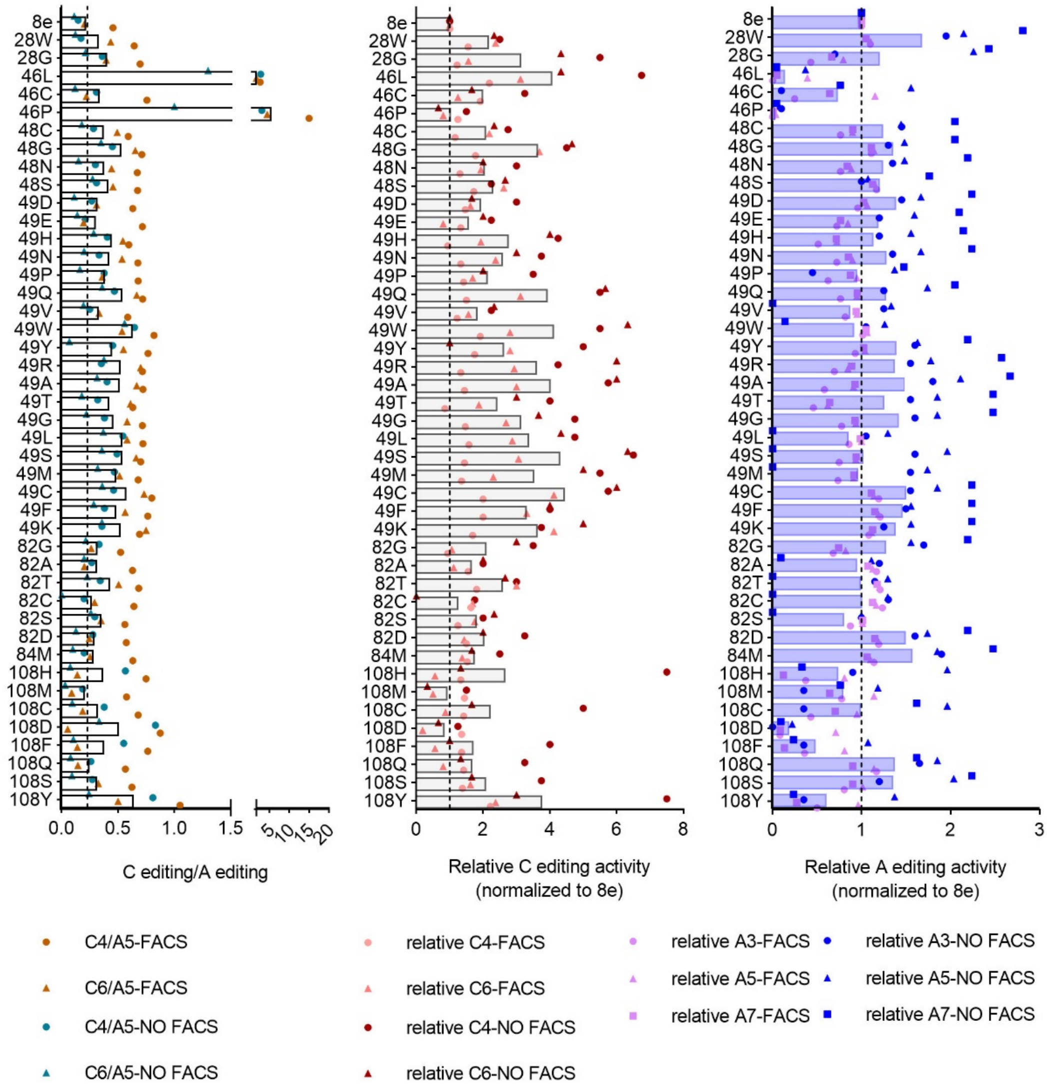

Supplementary Figure 10. Single mutations enhancing the C-to-T activity and preference of 49-NL-8e. Ratio of C-to-T at C4 and C6 to A-to-G activity at A5 (abbreviated as C4/A5 and C6/A5), and the relative C-to-T activity at C4, C6 and A-to-G editing activity at A3, A5, A7 normalized to 49-NL-8e were shown in bar graphs from right to left. Two biologically independent screening experiments were performed, with FACS and NO FACS representing screening with or without flow-cytometry enrichment. Source data are provided as a Source Data file.

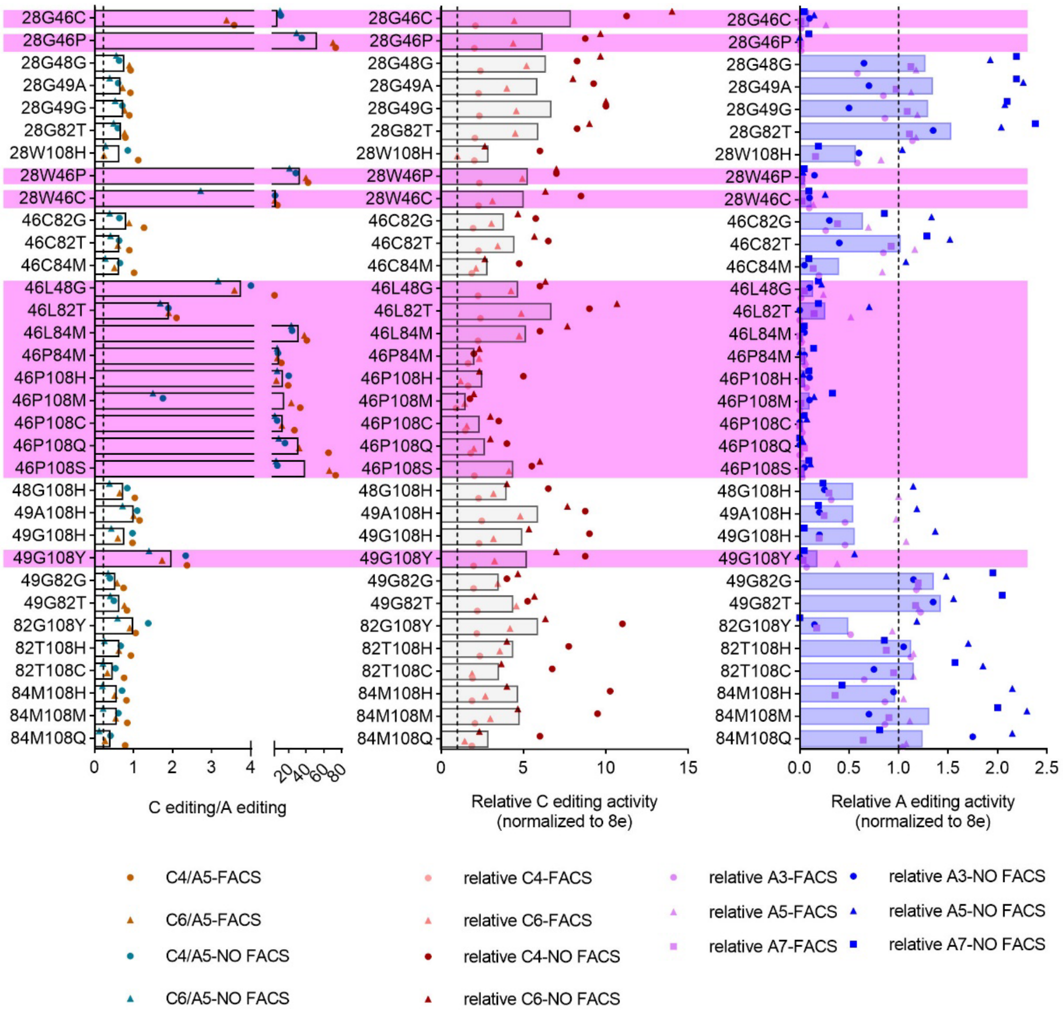

Supplementary Figure 11. Double mutations enhancing C-to-T activity and preference as compared to corresponding single mutations. Ratio of C-to-T at C4 and C6 to A-to-G activity at A5 (abbreviated as C4/A5 and C6/A5), and the relative C-to-T activity at C4, C6 and A-to-G editing activity at A3, A5, A7 normalized to 49-NL-8e were shown in bar graphs from right to left for double mutations enhancing C-to-T activity and preference as compared to corresponding single mutations. Double mutations with minimized adenine editing activity are highlighted in pink boxes. Two biologically independent screening experiments were performed, with FACS and NO FACS representing screening with or without flow-cytometry enrichment. Source data are provided as a Source Data file.

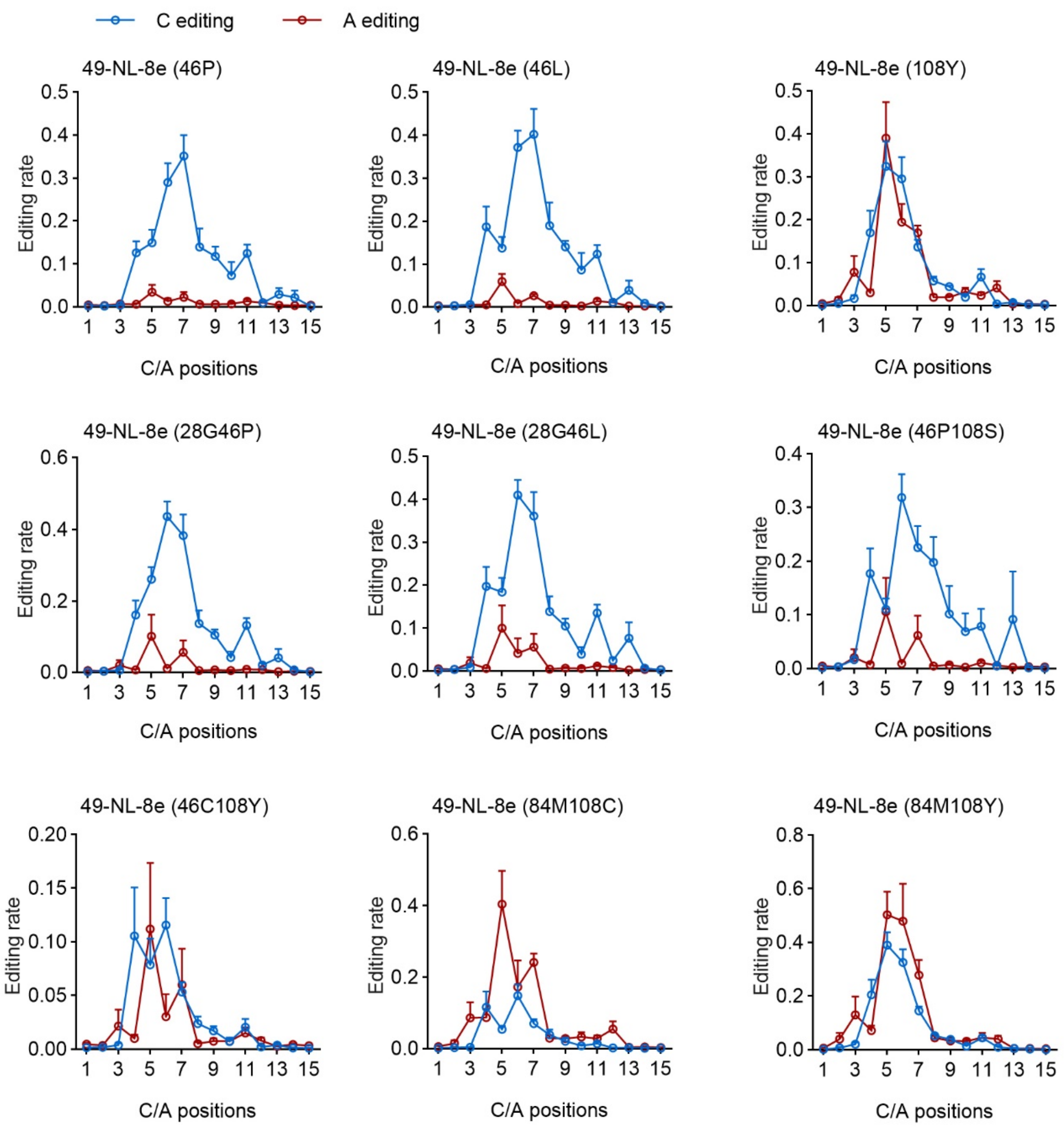

Supplementary Figure 12. Editing signatures of selected 49-NL-8e variants. Substrate specificities and editing scopes of other 49-NL-8e variants, including 49-NL-8e (46P), 49-NL-8e (46L), 49-NL-8e (108Y), 49-NL-8e (28G46P), 49-NL-8e (28G46L), 49-NL-8e (46P108S), 49-NL-8e (46C108Y), 49-NL-8e (84M108C) and 49-NL-8e (84M108Y) were shown. Adenine editing (A editing) was shown in red lines and cytosine editing (C editing) was shown in blue lines. Data was collected from three independent experiments and presented as mean ± SEM. in histograms. Source data are provided as a Source Data file.

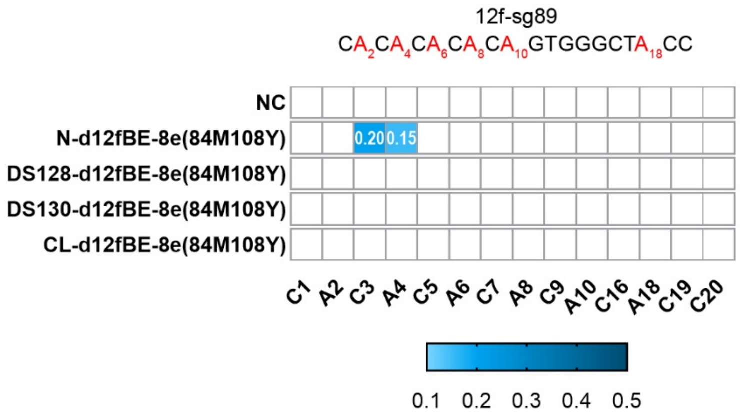

Supplementary Figure 13. Editing signatures of d12fBEs-8e (84M108Y). The d12fBEs were generated by fusing TadA-8e (84M108Y) to N-/C-terminus or 128/130 internal sites of d12f. Editing signatures were evaluated against sgRNA12f-sg89 and shown in heatmap in blue gradient color. Data in heatmap was collected from three independent experiments. NC, negative control. Source data are provided as a Source Data file.

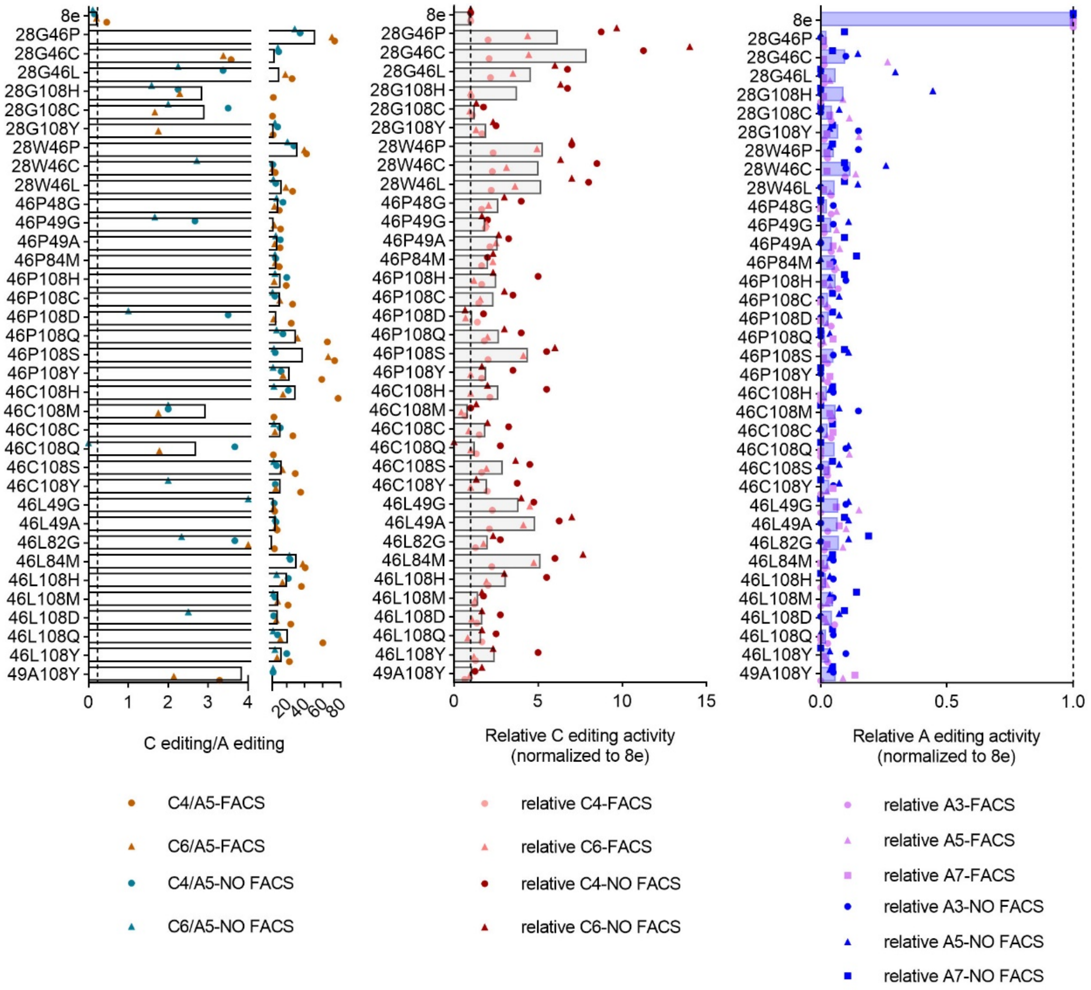

Supplementary Figure 14. Double mutations with minimized A-to-G activity and comparable or higher C-to-T activity. Bar graphs from left to right show ratios of C-to-T to A-to-G activity, relative C-to-T and A-to-G efficiencies normalized to 49-NL-8e, respectively. Mutations shown here displayed minimized A-to-G activity and comparable or higher C-to-T activity as compared to 49-NL-8e. Two biologically independent screening experiments were performed, with FACS representing screening with flow-cytometry enrichment, and NO FACS representing screening without flow-cytometry enrichment. Source data are provided as a Source Data file.

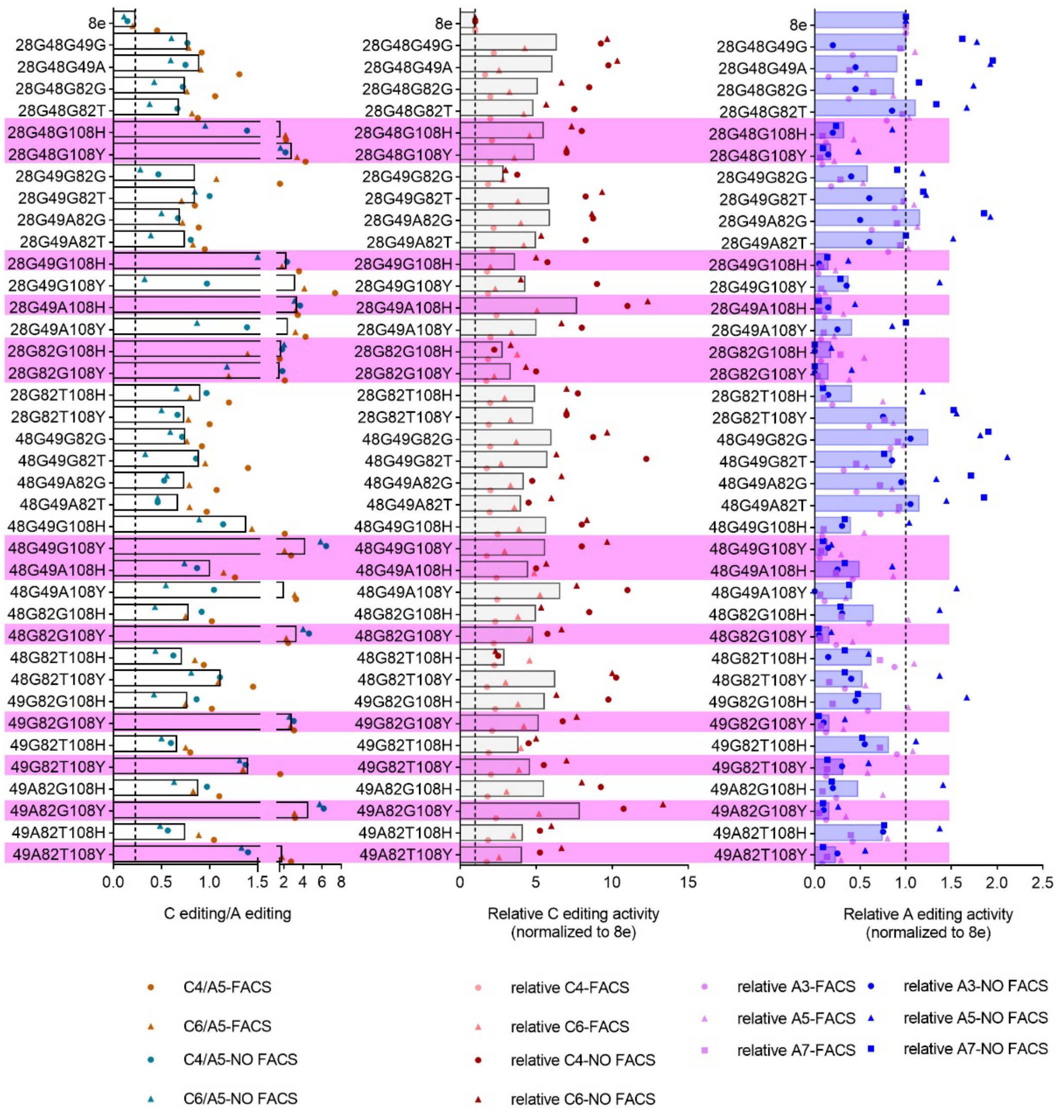

Supplementary Figure 15. Influence of triple mutations on cytosine and adenine editing activities. Ratio of C-to-T at C4 and C6 to A-to-G activity at A5 (abbreviated as C4/A5 and C6/A5), and the relative C-to-T activity at C4, C6 and A-to-G editing activity at A3, A5, A7 normalized to 49-NL-8e were shown in bar graphs from right to left for all triple mutation combinations of V28G, A48G, I49G/A, V82G/T and N108H/Y. Triple mutations with reduced A-to-G activity at all three sites A3, A5 and A7 are highlighted in pink boxes. Two biologically independent screening experiments were performed, with FACS and NO FACS representing screening with or without flow-cytometry enrichment. Source data are provided as a Source Data file.

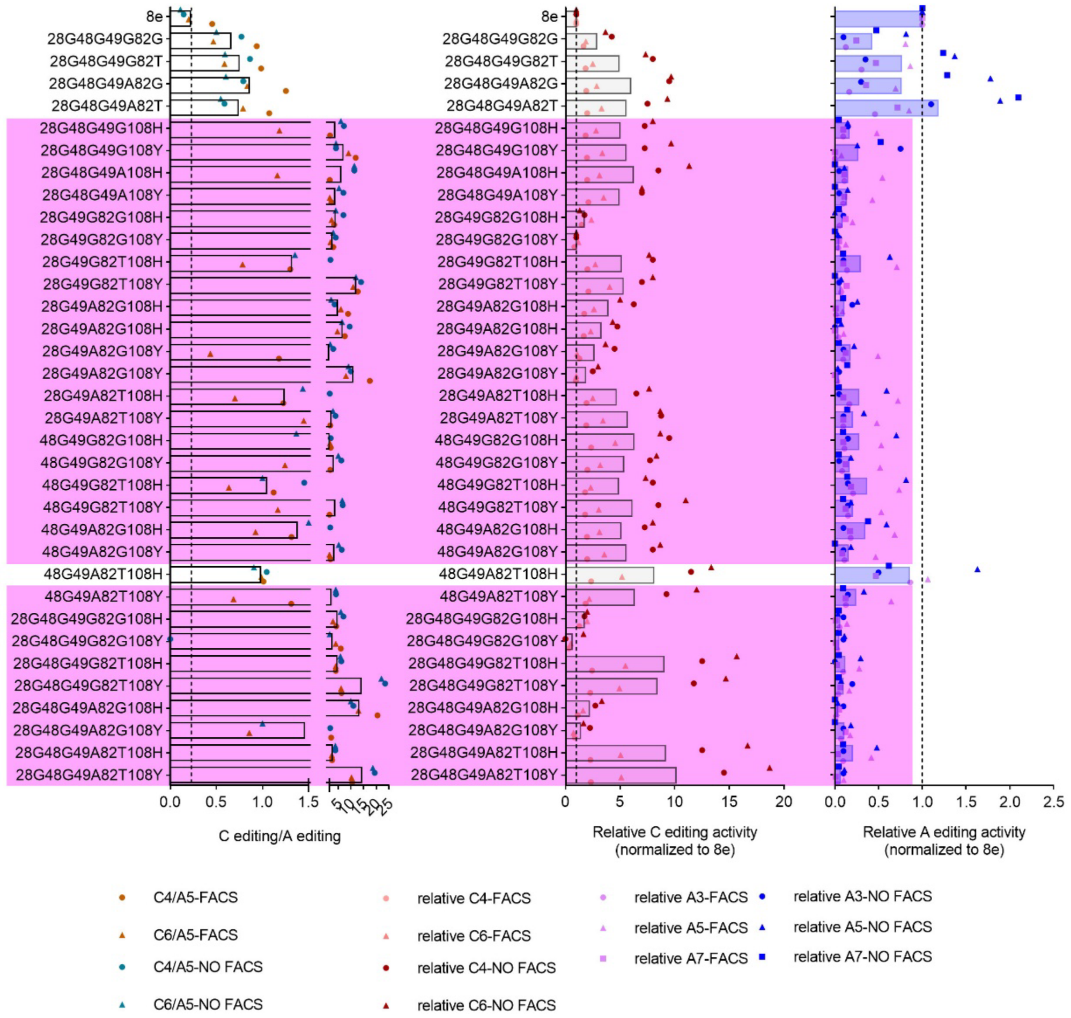

Supplementary Figure 16. Influence of quadruple and quintuple mutations on C-to-T and A-to-G activities. Ratio of C-to-T at C4 and C6 to A-to-G activity at A5 (abbreviated as C4/A5 and C6/A5), and the relative C-to-T activity at C4, C6 and A-to-G editing activity at A3, A5, A7 normalized to 49-NL-8e were shown in bar graphs from right to left for all quadruple and quintuple mutation combinations of V28G, A48G, I49G/A, V82G/T and N108H/Y. quadruple and quintuple mutation with reduced A-to-G activity at all three sites A3, A5 and A7 are highlighted in pink boxes. Two biologically independent screening experiments were performed, with FACS and NO FACS representing screening with or without flow-cytometry enrichment. Source data are provided as a Source Data file.

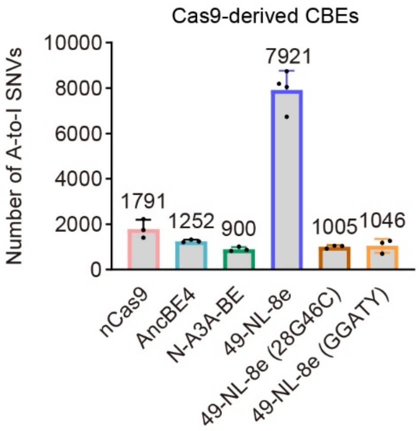

Supplementary Figure 17. A-to-I RNA off-target edits of representative TadA-reprogrammed CBEs. Number of adenine-to-inosine (A-to-I) RNA edits for classical CBEs AncBE4, N-A3A-BE and TadA-reprogrammed CBEs 49-NL-8e (28G46C), 49-NL-8e (GGATY). n=3 or 4 (49-NL-8e) biologically independent experiments, and presented as mean ± SEM. in histograms. Source data are provided as a Source Data file.

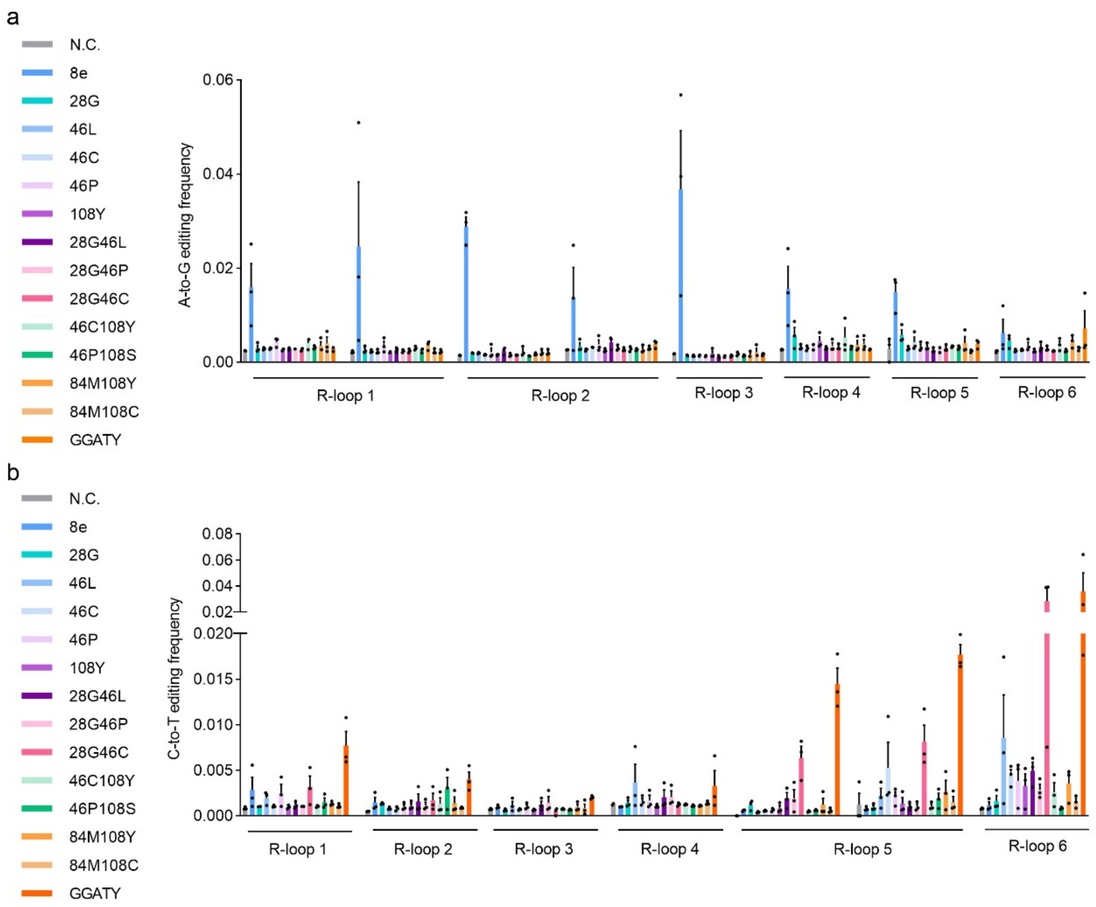

Supplementary Figure 18. Cas9-independent DNA off-target effects for selected 49-NL-8e variants analyzed with R-loop assay. R-loop assays at 6 sites were performed to assess the Cas9-independent DNA off-target effects, as shown by adenine-to-guanine (A-G) **(a)** and cytosine to thymine (C-T) frequencies **(b)**. Data was collected from three independent experiments and presented as mean ± SEM. in histograms. NC, negative control. Source data are provided as a Source Data file.

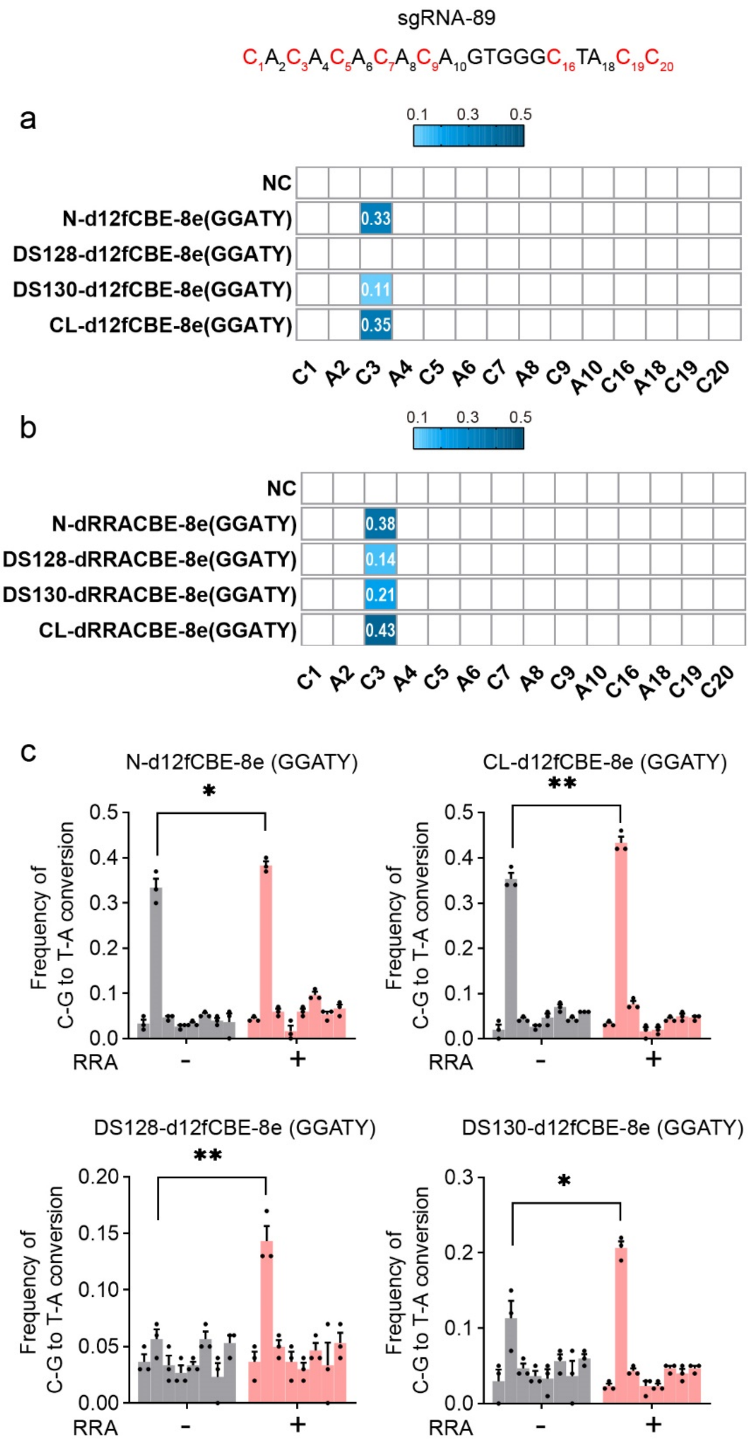

Supplementary Figure 19. Enhancement of editing activities for TadA-reprogrammed d12fCBEs by RRA mutations. a, Heatmap showing editing activities of d12fCBEs-8e (GGATY) against sgRNA 12f-sg89 without RRA mutations. **b,** Heatmap showing editing activities of d12fCBEs-8e (GGATY) against sgRNA 12f-sg89 with RRA mutations in blue gradient color. **c,** Histogram showing C-to-T editing

comparison of TadA-reprogrammed d12fCBEs-8e (GGATY) with or without RRNA mutations against sgRNA 12f-sg89. Data was collected from at least three independent experiments and presented as mean ± SEM. in histograms. *p<0.05, **p<0.01 with two-tailed unpaired t-test. n=3 biologically independent experiments. Exact P values were as follows, P=0.016, 0.00747, 0.00561, 0.02. NC, negative control. Source data are provided as a Source Data file.

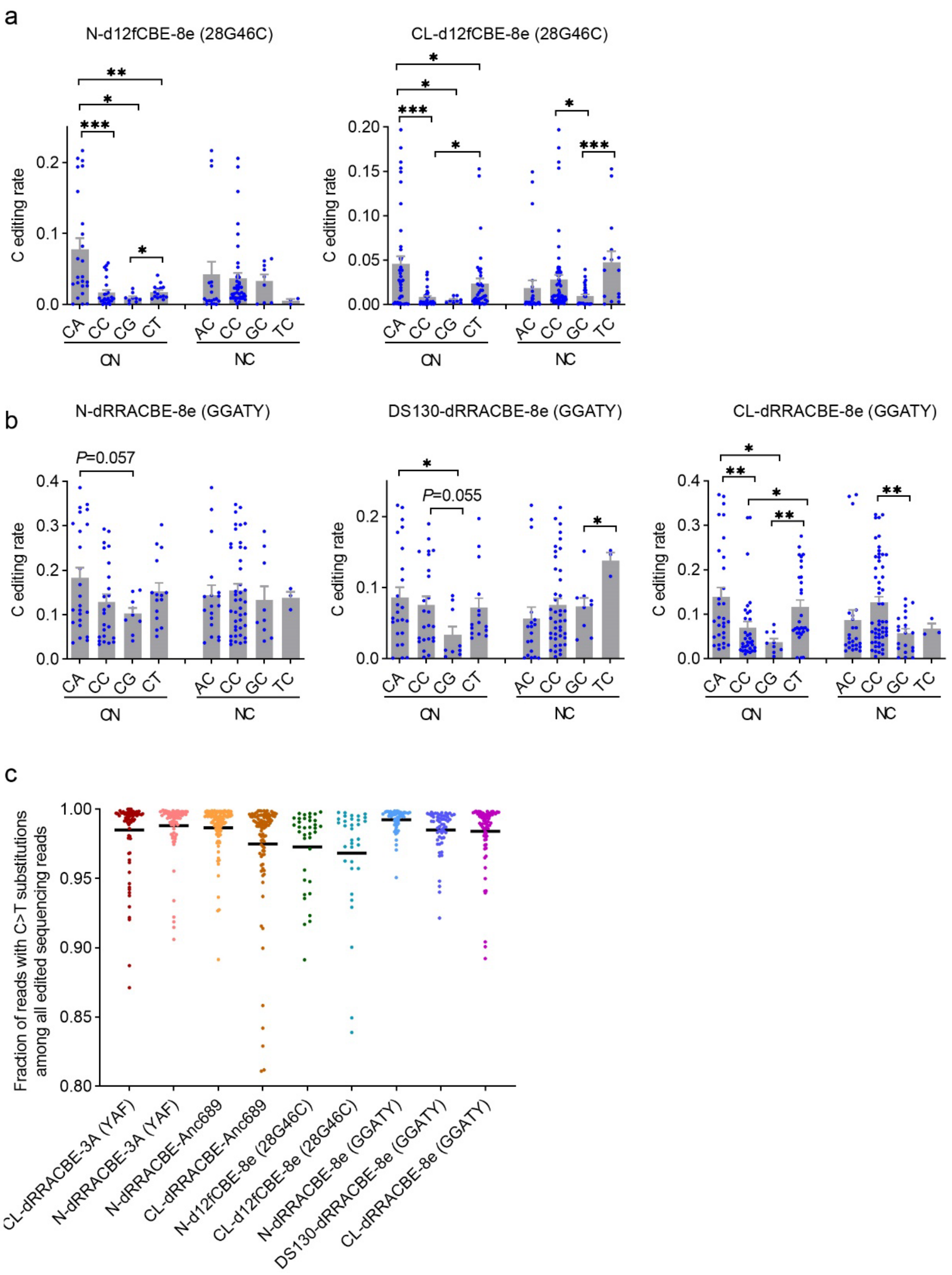

Supplementary Figure 20. Context preference and product purity of representative d12fCBEs. a-b, Context preference (NCN) was analyzed for TadA-reprogrammed N-/CL-d12fCBE-8e (28G46C) (**a**) and N-/DS130-/CL-d12fCBE-8e (GGATY) (**b**). **c,** Cytosines accessible to corresponding d12fCBEs and displaying obvious editing efficiencies were included for product purity analysis. Data was

presented as mean ± SEM. in histograms. *$p<0.05$, **$p<0.01$, ***$p<0.001$ with two-tailed unpaired t-test. n=3 biologically independent experiments. Exact *P* values were provided in Source data. Source data are provided as a Source Data file.

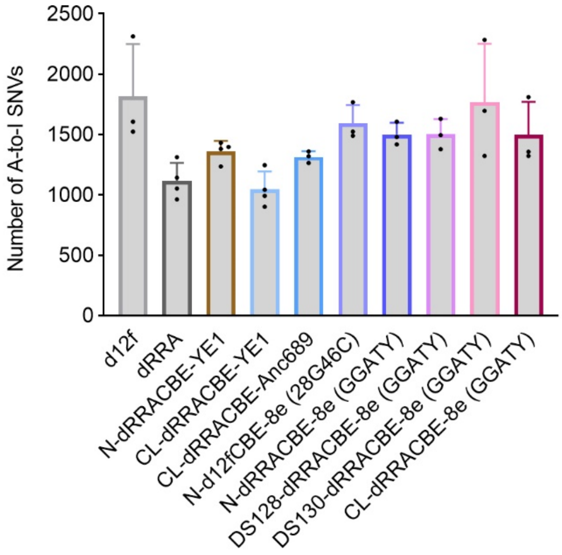

Supplementary Figure 21. A-to-I RNA off-target edits of representative d12fCBEs.

Number of A-to-I RNA edits for cytidine deaminase-derived d12fCBEs and TadA-reprogrammed d12fCBEs N-d12fCBE-8e (28G46C) and N-/DS128-/DS130-/CL-Drracbe-8e (GGATY). n=3 biologically independent experiments, and presented as mean ± SEM. in histograms. Source data are provided as a Source Data file.

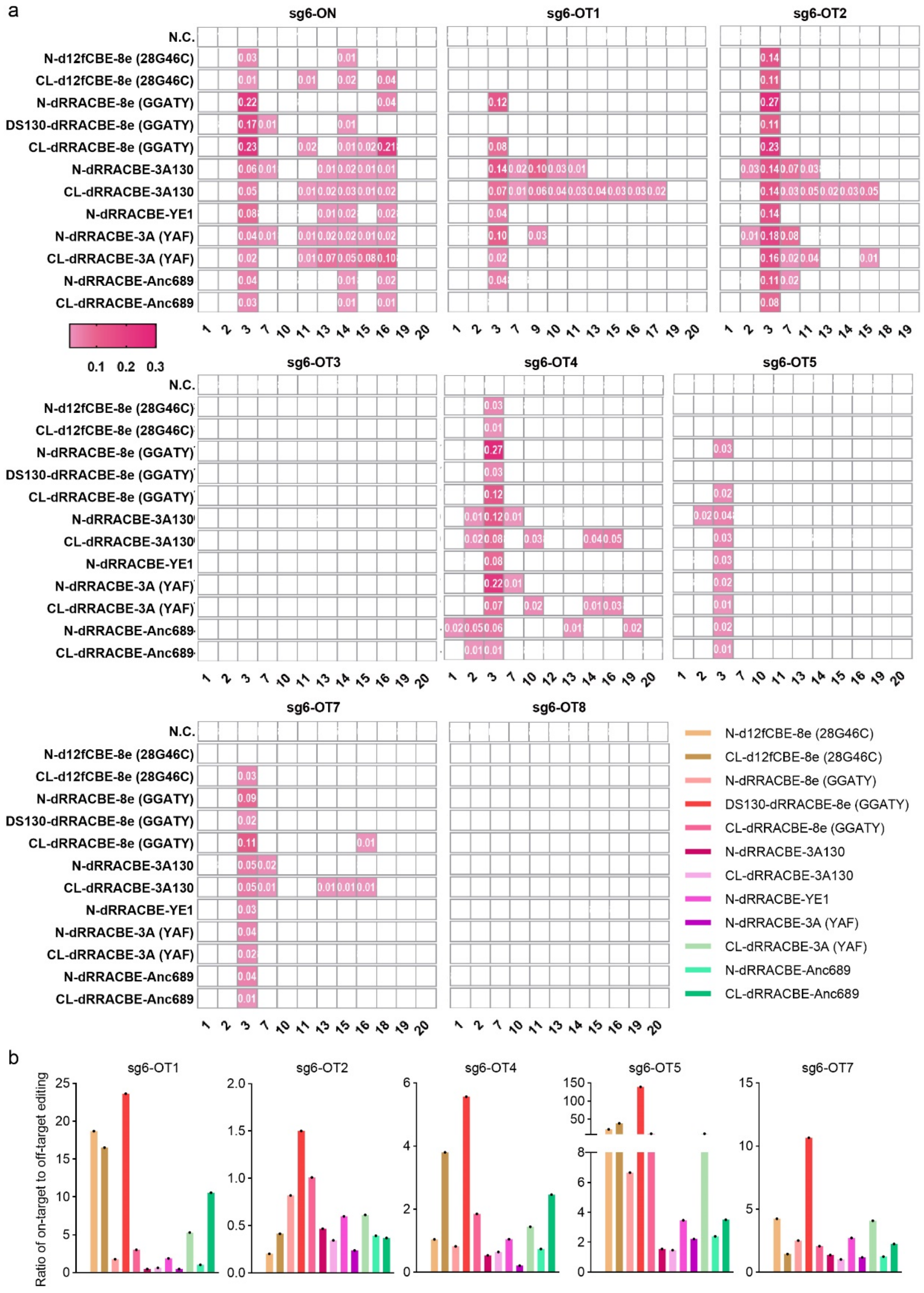

Supplementary Figure 22. Cas-dependent DNA off-target effects of representative d12fCBEs at 12f-sg6 site. a, Heatmaps showing the C-to-T editing activities of representative d12fCBEs at 12f-sg6 site and 8 potential off-target sites in pink gradient

color. **b,** On-to-off-target ratios of the top five potential off-target sites were quantified as follows: the highest on-target editing frequency/the highest off-target editing frequency. Data was collected from three independent experiments. NC, negative control. Source data are provided as a Source Data file.

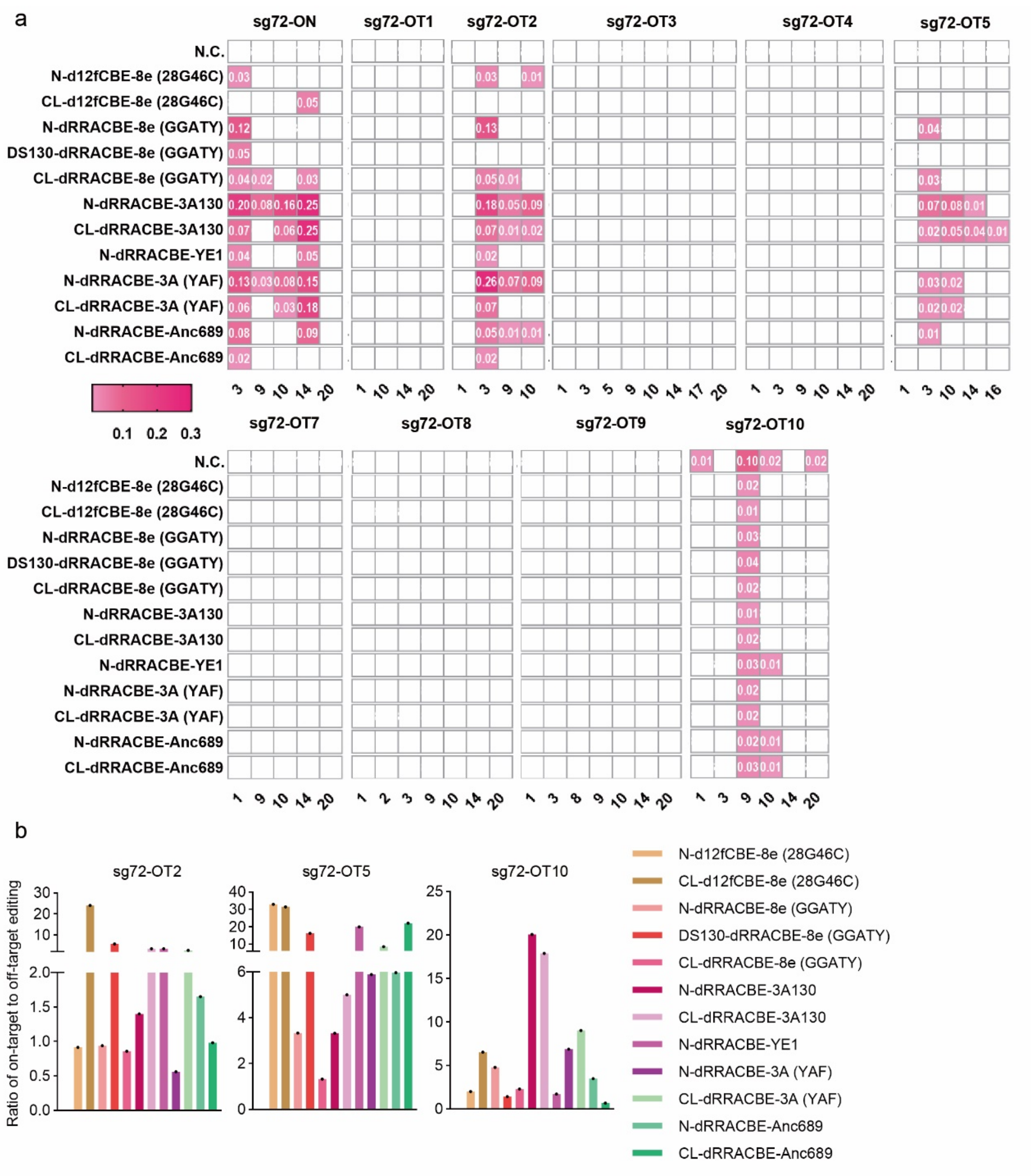

Supplementary Figure 23. Cas-dependent DNA off-target effects of representative d12fCBEs at 12f-sg72 site. a, Heatmaps showing the C-to-T editing activities of representative d12fCBEs at 12f-sg72 site and 9 potential off-target sites in pink gradient color. **b,** On-to-off-target ratios of the top three potential off-target sites were quantified as follows: the highest on-target editing frequency/the highest off-target editing frequency. Data was collected from three independent experiments. NC, negative control. Source data are provided as a Source Data file.

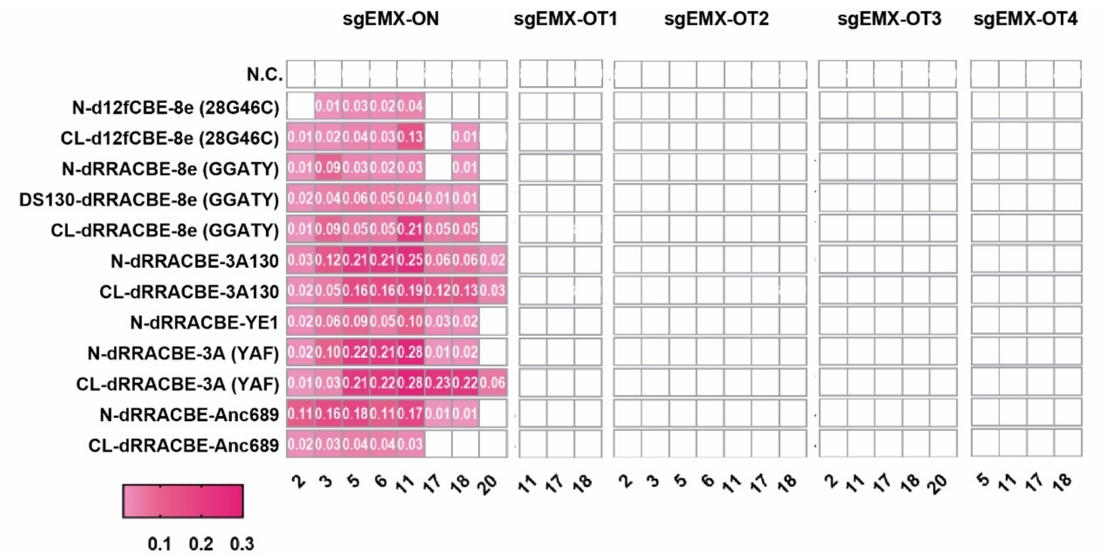

Supplementary Figure 24. Cas-dependent DNA off-target effects of representative d12fCBEs at 12f-sgEMX site. Heatmaps showing the C-to-T editing activities of representative d12fCBEs at 12f-sgEMX site and 4 potential off-target sites in pink gradient color. Data was collected from three independent experiments. NC, negative control. Source data are provided as a Source Data file.

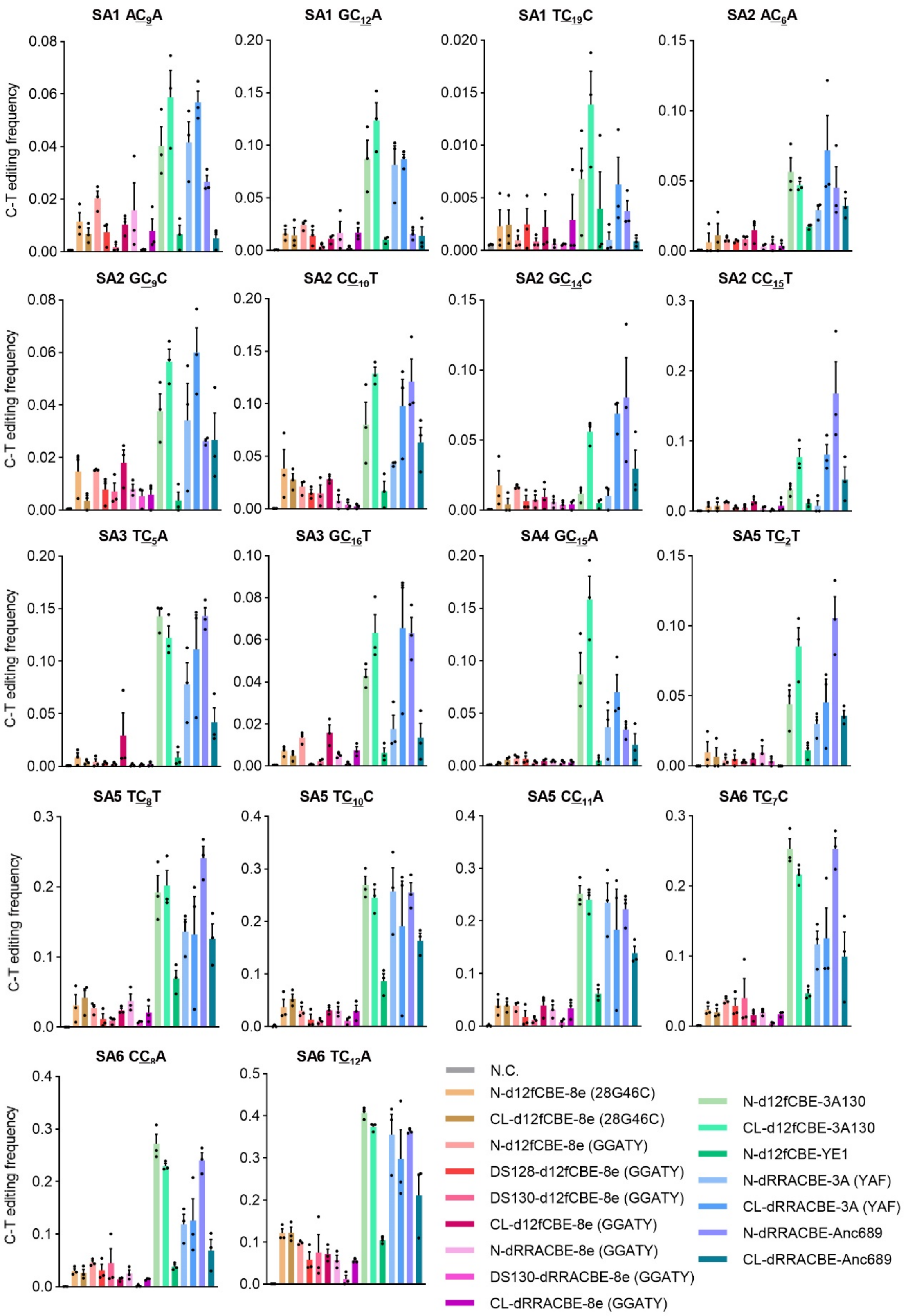

Supplementary Figure 25. Cas-independent DNA off-target effects of d12fCBEs. Cas-independent off-target C-to-T editing frequency of representative d12fCBEs were analyzed by R-loop assay. Data was collected from three independent experiments and

presented as mean ± SEM. in histograms. NC, negative control. Source data are provided as a Source Data file.

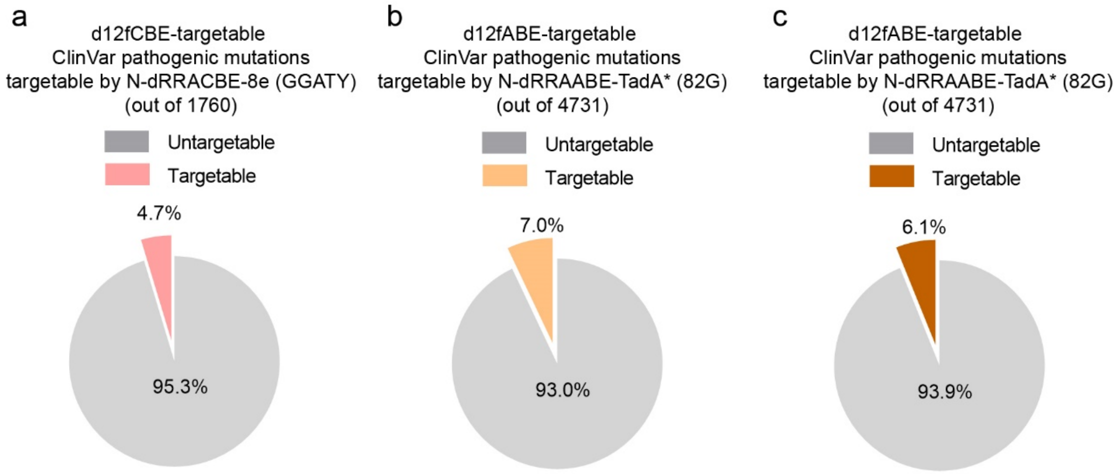

Supplementary Figure 26. Therapeutic potential of representative d12fCBEs and d12fABEs. a, Frequencies of d12fCBE-targetable genetic mutations in ClinVar that could be corrected precisely by N-dRRACBE-8e (GGATY) in principle. **b,** Frequencies of d12fABE-targetable genetic mutations in ClinVar that could be corrected precisely by N-dRRAABE-TadA* (82G) in principle. **c,** Frequencies of d12fABE-targetable genetic mutations in ClinVar that could be corrected precisely by CL-Drraabe-TadA* (82G) in principle. Source data are provided as a Source Data file.

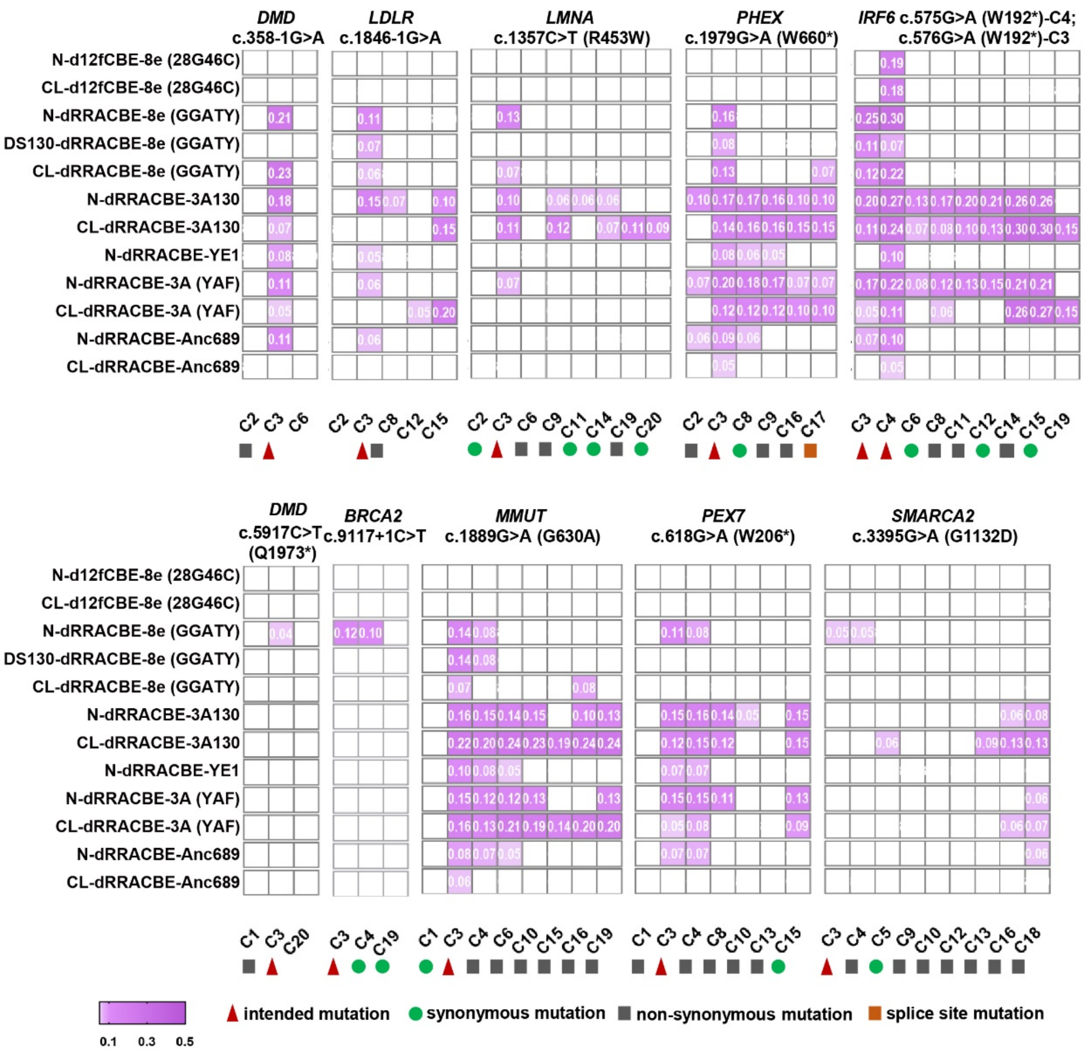

Supplementary Figure 27. Induction of pathogenic mutations with representative d12fCBEs in HEK293T cells. Ten different pathogenic mutations in ClinVar were selected and corresponding sgRNAs were designed. Then sgRNAs were co-transfected with specific d12fCBEs in HEK293T cells for 72h incubation. Positive cells were collected with flow cytometry and targeted amplicon sequencing was performed. Heatmaps showing the C-to-T editing frequencies at sites targeted by corresponding sgRNAs in purple gradient color. Data was collected from three independent experiments. Source data are provided as a Source Data file.

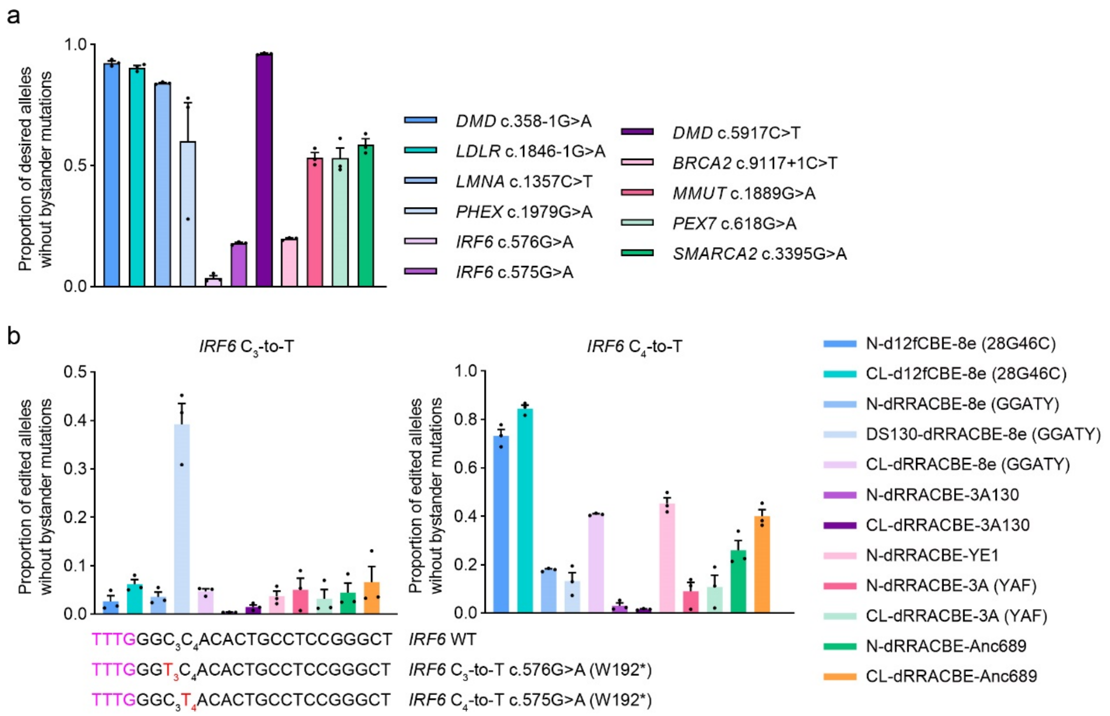

Supplementary Figure 28. Homogeneity of edited products for representative d12fCBEs. a, Proportion of desired edited alleles without bystander mutations induced by N-dRRACBE-8e (GGATY) for 11 pathogenic mutations. **b,** Proportion of desired edited alleles without bystander mutations induced by representative d12fCBEs at *IRF6* loci. C3 and C4 at *IRF6* loci (corresponding to c. 576G and c. 575G, respectively) were targetable to representative d12fCBEs and expected to generate c. 575G>A and c. 576G>A substitutions mimicking human pathogenic mutations. Proportion of edited alleles without bystander mutations was calculated as follows: reads of edited alleles only containing desired mutation/reads of edited alleles containing desired mutation with/without bystander mutations across sgRNA-targeting regions. Data was collected from three independent experiments and presented as mean ± SEM. in histograms. Source data are provided as a Source Data file.

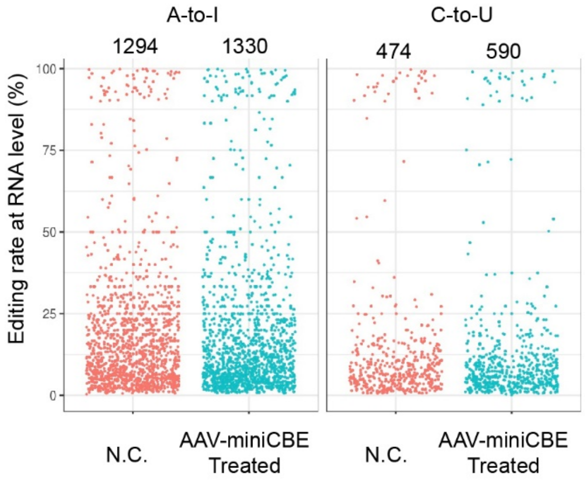

Supplementary Figure 29. RNA off-target editing in mice brain tissues treated with TadA-reprogrammed miniCBE N-dRRACBE-8e (GGATY) delivered by AAV9 system. NC, negative control.

Negative control

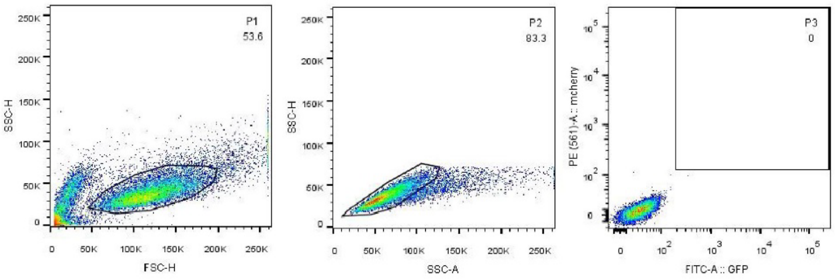

P1 P2 P3

Name	Statistic	#Cells
293T_N-1_008.fcs		20000
P1	53.6	10714
P2	83.3	8926
P3	0	0

mCherry

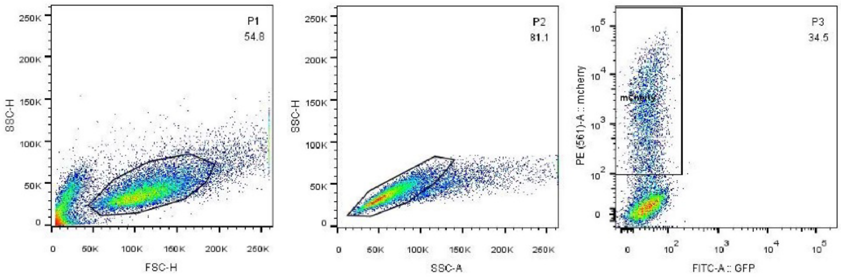

P1 P2 P3

Name	Statistic	#Cells
293T_mCherry-1_005.fcs		20000
P1	54.8	10966
P2	81.1	8895
P3	34.5	3072

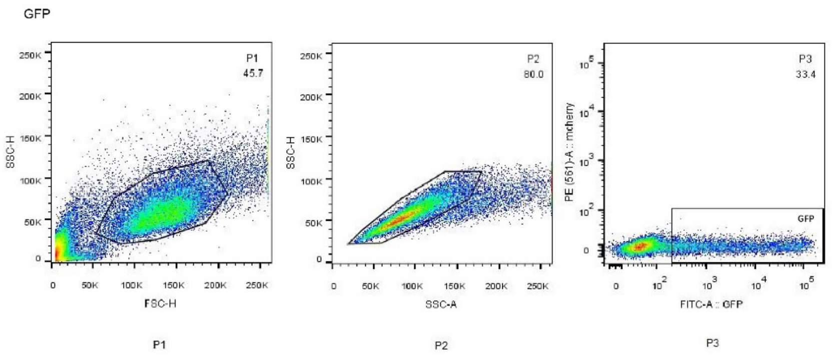

Name	Statistic	#Cells
293T_3_003.fcs		43430
P1	45.7	19847
P2	80.0	15878
P3	33.4	5301

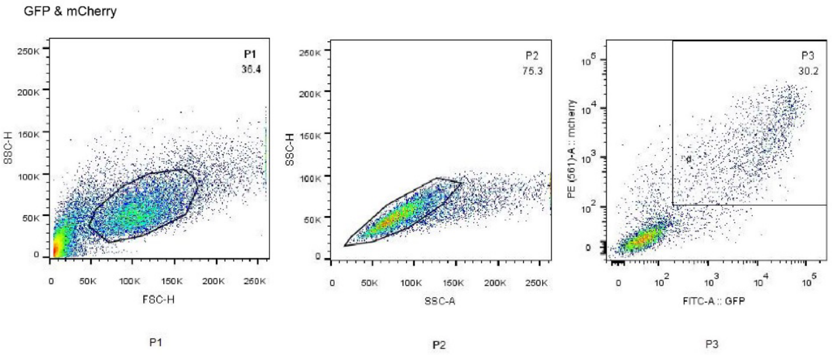

Name	Statistic	#Cells
293T_GR_003_004.fcs		20000
P1	36.4	7282
P2	75.3	5486
P3	30.2	1658

Supplementary Figure 30. Representative FACS gating figures for Negative control, mCherry, GFP and double positive samples. Samples for on-target base editing activity analysis and R-loop assays were collected as double positive gating/sorting panels.
