# Supplementary material for: TadA reprogramming to generate potent miniature base editors with high precision
Source: Nat Commun. 2023 Jan 26;14:413. doi: 10.1038/s41467-023-36004-2 (PMC9879996; doi:10.1038/s41467-023-36004-2)
Supplement: Supplementary file 2 — Description of Additional Supplementary Files [file 41467_2023_36004_MOESM2_ESM.pdf]

Title: Supplementary Data 1
Description: Information for Cas12f and sgRNA scaffold ge4.1

Title: Supplementary Data 2
Description: Detailed sgRNAs used in this study

Title: Supplementary Data 3
Description: all related primers for molecular cloning

Title: Supplementary Movie 1
Description: Potential internal docking sites of Cas12f1 for deaminase fusing
