# Supplementary material for: TadA reprogramming to generate potent miniature base editors with high precision
Source: Nat Commun. 2023 Jan 26;14:413. doi: 10.1038/s41467-023-36004-2 (PMC9879996; doi:10.1038/s41467-023-36004-2)
Supplement: Supplementary file 4 — Reporting Summary [file 41467_2023_36004_MOESM4_ESM.pdf]

nature portfolio

Corresponding author(s): Tian-Lin Cheng

Last updated by author(s): Jan 5, 2023

# Reporting Summary

Nature Portfolio wishes to improve the reproducibility of the work that we publish. This form provides structure for consistency and transparency in reporting. For further information on Nature Portfolio policies, see our Editorial Policies and the Editorial Policy Checklist.

## Statistics

For all statistical analyses, confirm that the following items are present in the figure legend, table legend, main text, or Methods section.

| n/a | Confirmed | |
|---|---|---|
| [ ] | [x] | The exact sample size (*n*) for each experimental group/condition, given as a discrete number and unit of measurement |
| [ ] | [x] | A statement on whether measurements were taken from distinct samples or whether the same sample was measured repeatedly |
| [ ] | [x] | The statistical test(s) used AND whether they are one- or two-sided<br>*Only common tests should be described solely by name; describe more complex techniques in the Methods section.* |
| [x] | [ ] | A description of all covariates tested |
| [x] | [ ] | A description of any assumptions or corrections, such as tests of normality and adjustment for multiple comparisons |
| [ ] | [x] | A full description of the statistical parameters including central tendency (e.g. means) or other basic estimates (e.g. regression coefficient) AND variation (e.g. standard deviation) or associated estimates of uncertainty (e.g. confidence intervals) |
| [ ] | [x] | For null hypothesis testing, the test statistic (e.g. *F*, *t*, *r*) with confidence intervals, effect sizes, degrees of freedom and *P* value noted<br>*Give P values as exact values whenever suitable.* |
| [x] | [ ] | For Bayesian analysis, information on the choice of priors and Markov chain Monte Carlo settings |
| [x] | [ ] | For hierarchical and complex designs, identification of the appropriate level for tests and full reporting of outcomes |
| [x] | [ ] | Estimates of effect sizes (e.g. Cohen's *d*, Pearson's *r*), indicating how they were calculated |

*Our web collection on statistics for biologists contains articles on many of the points above.*

## Software and code

Policy information about availability of computer code

| | |
|---|---|
| Data collection | Deep sequencing for amplicon products was performed using Illumina NovaSeq platform at Shanghai Personalbio Technology, and n = 3 or 4 independent biological replicates were performed with the mean ± s.e.m. shown in figures.<br>MRNA-seq data following the long-RNA-seq-pipeline of ENCODE Consortium. Reads were aligned to GRCh38 reference genome using annotation GRCh38.v96 via STAR (v2.4.2a) in 2-pass mode 46. Gene counts were quantified using RSEM (v1.3.3) 47. Variant calling from RNA-seq data was performed using Sentieon genomics tools (v202010.02). Sequenced reads were first mapped to the reference genome (GRCh38) with STAR (v2.4.2a) in two-pass mode as described above. Picard (v2.23.6, http://broadinstitute.github.io/picard) was used to sort and mark duplicates of the mapped BAM files. After removing duplicates, reads were split at junctions into exon segments and reassigned the mapping qualities from STAR. Base quality score recalibration (BQSR) was performed as the DNA-seq to remove experimental biases caused by the sequencing methodology. Then, variants were identified by MuTect2 tool 25, 48. To identify high confidence variants, we performed hard-filtering using VariantFiltration tool of GATK with parameter --filter-expression 'QUAL <25 \|\| MQ < 20.0 \|\| QD < 2.0 \|\| FS > 30.0 \|\| DP < 20' to filter variants with base-quality score < 25, mapping quality score < 20, Fisher strand values > 30.0, qual by depth values < 2.0 or sequencing depth < 20.<br>WGSwas performed using MGIseq platform (Genewiz). Paired-end sequencing reads were aligned to human genome build 38 (GRCh38/hg38) using BWA-MEM(v.0.7.17)44. Then bams were sorted, optical duplicates were marked and base quality recalibration was performed with GATK (v.4.2.4) tools MarkIlluminaAdapters, SamToFastq, MergeBamAlignment, SortSam, MarkDuplicates, SetNmMdAndUqTags, BaseRecalibrator and ApplyBQSR. High-performance computing cluster was used for subsequent analyses. Google DeepVariant with default parameter45 was used for variant calling. Variant statistics were performed using custom shell scripts and Excel. |
| Data analysis | PCR products were analyzed using Sanger sequencing (BGI, Shenzhen, China) with EditR (v10) software.<br>Targeted amplicon sequencing (Shanghai Personalbio Technology, Shanghai, China) data including editing scope analysis and Cas-dependent and -independent DNA off-target analysis was performed with CRISPResso2. |

RNA-seq data was analyzed with following: RSEM (v1.3.3). Sentieon genomics tools (v202010.02). STAR (v2.4.2a).Picard (v2.23.6, http://broadinstitute.github.io/picard).MuTect2(202010.02).VariantFiltration tool (gatk-4.1.4.0).
Whole-genome-sequencing was analyzed with BWA-MEM(v.0.7.17). GATK (v.4.2.4) tools MarkIlluminaAdapters, SamToFastq, MergeBamAlignment, SortSam, MarkDuplicates, SetNmMdAndUqTags, BaseRecalibrator and ApplyBQSR. Google DeepVariant, and Excel 2016.

For manuscripts utilizing custom algorithms or software that are central to the research but not yet described in published literature, software must be made available to editors and reviewers. We strongly encourage code deposition in a community repository (e.g. GitHub). See the Nature Portfolio guidelines for submitting code & software for further information.

## Data

Policy information about availability of data

All manuscripts must include a data availability statement. This statement should provide the following information, where applicable:

- Accession codes, unique identifiers, or web links for publicly available datasets
- A description of any restrictions on data availability
- For clinical datasets or third party data, please ensure that the statement adheres to our policy

Raw high-throughput sequencing data are available in the NCBI sequence Read Archive database (PRJNA758206, PRJNA757902). Other additional relevant data are available upon reasonable request.

## Human research participants

Policy information about studies involving human research participants and Sex and Gender in Research.

| | |
|---|---|
| Reporting on sex and gender | not applicable |
| Population characteristics | not applicable |
| Recruitment | not applicable |
| Ethics oversight | not applicable |

Note that full information on the approval of the study protocol must also be provided in the manuscript.

# Field-specific reporting

Please select the one below that is the best fit for your research. If you are not sure, read the appropriate sections before making your selection.

[x] Life sciences [ ] Behavioural & social sciences [ ] Ecological, evolutionary & environmental sciences

For a reference copy of the document with all sections, see nature.com/documents/nr-reporting-summary-flat.pdf

# Life sciences study design

All studies must disclose on these points even when the disclosure is negative.

| | |
|---|---|
| Sample size | No statistical analysis were performed to predetermine sample size. but our sample sizes are in accordance to those generally used in this field. For TadA mutagenesis screening, 2 independent biological replicates were performed. For all other experiments performed in cell lines, at least 3 biological replicates were performed. |
| Data exclusions | No data was excluded. |
| Replication | Biological replicates and triplicates were performed independently. All attempts at replication were successful. |
| Randomization | Cells used in this study were grown under identical conditions, no randomization was used. Mice used for intraventricular injection were allocated to control or AAV9-treated group randomly. |
| Blinding | Blinding was not used, because no subjective assessments were required. |

# Reporting for specific materials, systems and methods

We require information from authors about some types of materials, experimental systems and methods used in many studies. Here, indicate whether each material, system or method listed is relevant to your study. If you are not sure if a list item applies to your research, read the appropriate section before selecting a response.

## Materials & experimental systems

| n/a | Involved in the study |
| --- | --- |
| x | Antibodies |
| | x Eukaryotic cell lines |
| x | Palaeontology and archaeology |
| | x Animals and other organisms |
| x | Clinical data |
| x | Dual use research of concern |

## Methods

| n/a | Involved in the study |
| --- | --- |
| x | ChIP-seq |
| | x Flow cytometry |
| x | MRI-based neuroimaging |

# Eukaryotic cell lines

Policy information about cell lines and Sex and Gender in Research

| | |
| --- | --- |
| Cell line source(s) | HEK293T cells (Cell Bank of the Chinese Academy of Sciences, Shanghai, China) |
| Authentication | Cells were authenticated by the supplier using STR analysis. |
| Mycoplasma contamination | Cells tested negative mycoplasma. |
| Commonly misidentified lines (See ICLAC register) | None commonly misidentified lines used. |

# Animals and other research organisms

Policy information about studies involving animals; ARRIVE guidelines recommended for reporting animal research, and Sex and Gender in Research

| | |
| --- | --- |
| Laboratory animals | The Postnatal day 0 (P0) C57BL/6 Mus musculus (Vital River Laboratories) was used in this study. According to animal welfare requirements, all experimental mice were bred on the condition of a 12h light/dark cycle (dark from 7pm to 7 am while light from 7 a.m. to 7 p.m.) with food/water provided ad libitum. Additionally, pathogen free (PF) unit under constant temperature (approximately 22 ℃), humidity (approximately 55%RH), ventilation and automatic circadian rhythm were maintained for experimental mice. |
| Wild animals | No wild animals were used |
| Reporting on sex | Sex was not considered in this study. |
| Field-collected samples | The study did not involve samples collected from the field. |
| Ethics oversight | Ethical Statement. Our research complies with all relevant ethical regulations, and animal experiments have been approved by and were in accordance with the guidelines of the Animal Committee of Department of Laboratory Science, Fudan University, China. |

Note that full information on the approval of the study protocol must also be provided in the manuscript.

# Flow Cytometry

## Plots

Confirm that:

- [x] The axis labels state the marker and fluorochrome used (e.g. CD4-FITC).
- [x] The axis scales are clearly visible. Include numbers along axes only for bottom left plot of group (a 'group' is an analysis of identical markers).
- [x] All plots are contour plots with outliers or pseudocolor plots.
- [x] A numerical value for number of cells or percentage (with statistics) is provided.

## Methodology

| | |
| --- | --- |
| Sample preparation | HEK293T cells were plated into 6-well or 48-well plates (Corning) 24 h before transfection. Then transfection was performed using Lipo293TM (Beyotime biotechnology, Shanghai, China) according to the manufacturer's instructions. Briefly, base editor-expressing plasmid and sgRNA-expressing plasmid (mole ratio 2:1) were mixed with 2.5~3 µl (48-well) or 8 µl (6-well) Lipo293TM and incubated at room time for 10 minutes before adding into the wells. Cells were cultured for another 72 h and collected for base editing analysis. For IscB experiments, cells were cultured for 96 hours after transfection for base editing analysis. |
| Instrument | Moflo XDP, Beckman Coulter/ BD FACSAria™ Fusion Flow Cytometers |

| | |
|---|---|
| Software | FlowJo X 10.0.7 |
| Cell population abundance | cell population abundances for target populations were similar across different base editor experiments. Transfected HEK293T cells usually were ~ 20-40% GFP/mCherry double positive. |
| Gating strategy | Gates were established using untransfected control cells, transfected GFP+ cells and transfected mCherry+ cells. Gates were drawn to collect all double positive cells. |

☒ Tick this box to confirm that a figure exemplifying the gating strategy is provided in the Supplementary Information.
